# Expression of SERPINA3s in cattle: focus on bovSERPINA3-7 reveals specific involvement in skeletal muscle

**DOI:** 10.1098/rsob.150071

**Published:** 2015-09-09

**Authors:** Antoine Péré-Brissaud, Xavier Blanchet, Didier Delourme, Patrick Pélissier, Lionel Forestier, Arnaud Delavaud, Nathalie Duprat, Brigitte Picard, Abderrahman Maftah, Laure Brémaud

**Affiliations:** 1 INRA, Université de Limoges, UMR1061 Génétique Moléculaire Animale, Limoges, France; 2 UMR1213 Herbivores, UMRH-AMUVI, INRA de Clermont Ferrand Theix, St Genès Champanelle, France

**Keywords:** serpin, α_1_-antichymotrypsin, protease inhibitor, skeletal muscle, bovine

## Abstract

α_1_-Antichymotrypsin is encoded by the unique *SERPINA3* gene in humans, while it is encoded by a cluster of eight closely related genes in cattle. BovSERPINA3 proteins present a high degree of similarity and significant divergences in the reactive centre loop (RCL) domains which are responsible for the antiprotease activity. In this study, we analysed their expression patterns in a range of cattle tissues. Even if their expression is ubiquitous, we showed that the expression levels of each serpin vary in different tissues of 15-month-old Charolais bulls. Our results led us to focus on bovSERPINA3-7, one of the two most divergent members of the bovSERPINA3 family. Expression analyses showed that bovSERPINA3-7 protein presents different tissue-specific patterns with diverse degrees of *N*-glycosylation. Using a specific antibody raised against bovSERPINA3-7, Western blot analysis revealed a specific 96 kDa band in skeletal muscle. BovSERPINA3-7 immunoprecipitation and mass spectrometry revealed that this 96 kDa band corresponds to a complex of bovSERPINA3-7 and creatine kinase M-type. Finally, we reported that the bovSERPINA3-7 protein is present in slow-twitch skeletal myofibres. Precisely, bovSERPINA3-7 specifically colocalized with myomesin at the M-band region of sarcomeres where it could interact with other components such as creatine kinase M-type. This study opens new prospects on the bovSERPINA3-7 function in skeletal muscle and promotes opportunities for further understanding of the physiological role(s) of serpins.

## 1. Introduction

The serine protease inhibitors (serpins) constitute a large family of functionally diverse proteins that are found in all kingdoms including animals, plants, bacteria and some viruses [1–3]. Although their physiological functions are still not fully elucidated, most serpins are involved in numerous intracellular and extracellular processes such as blood coagulation, fibrinolysis, cell migration or tumour suppression [4,5]. Moreover, some studies have reported the association between serpins and numerous familial disorders or diseases known as serpinopathies [6,7]. In those cases, serpins act by forming large and stable multimers. Such conformational disorders are known for thrombosis induced by mutations in antithrombin [8], emphysema or liver cirrhosis induced by mutations in *α*_1_-antitrypsin [9,10], or dementia caused by polymerization and tissue deposition of the mutated neuroserpin [11]. In addition to the known protease inhibitory function of serpins [5,12], some members have evolved and acquired non-inhibitory roles in diverse events such as blood pressure regulation (angiotensinogen), chromatin condensation (protein MENT) or hormone transport (corticosteroid binding globulin, CBG and thyroxine binding globulin, TBG) [12,13]. In *Drosophila*, the serpin Spn27A is implicated in the formation of the dorsoventral axis during embryonic development [14]. In CBG and TBG cases, the decrease of hormone affinity results from the spontaneous insertion of reactive centre loop (RCL) domain into the β-sheet A upon cleavage. In the other cases, the RCL seems to be not functional, and it appears that one or more regions apart from the RCL are involved in biological activities of the serpins.

Despite their diversity of function(s), these proteins share a conserved 350-residue core globular domain consisting of three β-sheets and eight or nine α-helices [15]. They present a short flexible strand, the RCL domain, which contains the recognition region for the target protease. Many serpins inhibit their targets by forming stable complexes with the proteolytic enzymes. This interaction is RCL-dependent and induces conformational changes as in the mechanism of suicide substrate inhibitor [3,12,16].

Currently, the NCBI protein database contains at least 20 000 entries annotated as serpins. According to their gene structures, the exon–intron-based system includes six groups of vertebrate serpins [17]. Based on sequence similarities, the serpin family is divided into nine first clades (A to I) [5]. So far, the two largest groups are the antitrypsin-like and the ovalbumin-like serpins, named ‘serpin A’ and ‘serpin B’, respectively. The *SERPINA* genes typically consist of four exons with identical positioning and phasing of intron–exon boundaries. Chromosomal localization studies have highlighted the complexity of the family and revealed that serpin genes are often clustered and share similarities in structure, presumably as a result of gene duplication [17].

Among the known serpins, SERPINA3, also called α_1_-antichymotrypsin (α_1_-ACT), is one of the most studied and abundant serpins that appears to be physiologically essential. The main role of this protein is mediated by its inhibitory effect against cathepsin G and chymotrypsin-like enzymes [18,19]. Although its biological functions are not fully elucidated, α_1_-ACT seems to play an important role in immune response modulation. After tissue injury or infection, *α*_1_-ACT concentration increases leading to the inhibition of the natural cytotoxic activity of T-cell killer lymphocytes and the enhancement of antibody response [20,21]. Mutations in *α*_1_-ACT induce the formation of inactive polymers that cause the protein retention within hepatocytes and plasma deficiency [22]. More recently, some polymorphisms in the *SERPINA3* gene have been associated with Alzheimer's disease [23].

In contrast to humans, where α_1_-ACT is represented by the single *SERPINA3* gene at position 14q32.1 [24], the *SERPINA3* locus on other mammalian species is dramatically expanded. The mouse multi-genic locus, termed Spi-2, is a cluster of 14 members with 65–85% similarity and a markedly divergent RCL domain [25]. In pig, three α_1_-ACTs are detected at the protein level: PI2 (SERPINA3-1), PI3 and PI4 (SERPINA3 paralogues); and an additional serpin, *SERPINA3-2*, is identified at the genomic level [26,27]. As observed in mice, pig PI2 and SERPINA3-2 proteins show 76% amino acid identity; the main difference resides near to the C-terminus region that includes the deduced RCL domain. More recently, we provided additional information about *SERPINA3* status in the bovine genome. We characterized, a cluster of eight genes and one pseudo-gene at the 21q24 position [28]. Interestingly, this cluster contains an original subgroup of six members (*bovSERPINA3-1* to *bovSERPINA3-6*) with an unexpectedly high degree of conservation (96%). This subgroup is not found in mouse and pig *SERPINA3* clusters. The two remaining genes (*bovSERPINA3-7* and *bovSERPINA3-8*) are more different (tables 1 and 2).

**Table 1.** Main characteristics of the members of the bovSERPINA3 family.

bovine serpines A3	signal peptide	chain	length	mass (Da) whole/processed	UniProt	*N*-glycosylation sites
bovSERPINA3-1	1–24	387	411	46 240/43 650	Q9TTE1	100/180/230/264
bovSERPINA3-2	1–24	387	411	46 240/ 43 650	A2I7M9	100/180/230/264
bovSERPINA3-3	1–24	387	411	46 330/43 780	Q3ZEJ6	100/180/230/264/318
bovSERPINA3-4	1–24	387	411	46 310/43 760	A2I7N0	180/230/264/318
bovSERPINA3-5	1–24	387	411	46 400/43 780	A2I7N1	100/180/230/264/318
bovSERPINA3-6	1–25	389	414	46 390/43 720	A2I7N2	103/183/233/267/321
bovSERPINA3-7	1–25	392	417	46 940/44 170	A2I7N3	103/183/221/267
bovSERPINA3-8	1–25	393	418	46 960/44 280	A6QPQ2	103/183/233/268

recombinant serpin A3	*N*-His tag peptide			mass (Da) whole protein		
recSERPINA3-3	1–23	387	410	46 540		
recSERPINA3-7	1–23	392	415	46 930

**Table 2.** Comparison of RCL sequences of the bovSERPINA3 family. The potential RCL within the C-terminus loop is responsible for the specificity of inhibition. It was identified for the eight putative bovSERPINA3s by analogy with other serpins. The arginine residue (R) in italics for bovSERPINA3-1 to bovSERPINA3-6 could be the P1 residue for trypsin cleavage. Numbering at the two extremities refers to positions in each full sequence. Dots indicate identical residues compared with those of bovSERPINA3-1 and dashes show gaps in the alignment (adapted from Pélissier *et al*. [28]).

bovSERPINA3-1	361	G	T	E	G	A	A	A	T	G	I	S	M	E	*R*	T	I	L	R	—	—	I	I	V	R	382
bovSERPINA3-2	361	·	·	·	·	V	·	·	·	·	·	G	I	·	·	·	F	·	·	—	—	·	·	·	·	382
bovSERPINA3-3	361	·	·	·	·	·	·	·	·	·	·	G	I	·	·	·	F	·	·	—	—	·	·	·	·	382
bovSERPINA3-4	361	·	·	·	·	·	·	·	·	·	·	G	I	·	·	·	F	·	·	—	—	·	·	·	·	382
bovSERPINA3-5	361	·	·	·	·	·	·	·	·	·	·	G	I	·	·	·	F	·	·	—	—	·	·	·	·	382
bovSERPINA3-6	364	·	·	·	·	·	·	·	·	·	·	G	I	·	·	·	F	·	·	—	—	·	·	·	·	385
bovSERPINA3-7	365	·	·	·	·	·	·	V	·	A	V	V	·	A	T	S	S	·	L	H	T	L	T	·	S	388
bovSERPINA3-8	366	·	·	·	·	·	·	·	·	·	V	K	V	G	I	T	S	I	N	N	H	·	P	L	S	389

One intriguing question in the field is to address the biological significances of the eight closely related SERPINA3 proteins in cattle. BovSERPINA3-1 and bovSERPINA3-7 (often referred to as endopin 1 and endopin 2, respectively) have been studied in neurosecretory vesicles of chromaffin cells [29,30]. The authors suggested that bovSERPINA3-7 inhibits both serine and cysteine proteases of the regulated secretory pathway in chromaffin cells [30,31]. Also, our molecular and biochemical characterizations showed that bovSERPINA3-1 and bovSERPINA3-3 are expressed in skeletal muscles [32,33] and are able to strongly inhibit the initiator caspase 8 and the effector caspase 3 [34]. Although their biological roles are still uncertain, it could be assumed that both bovSERPINA3-1 and bovSERPINA3-3 are significantly relevant *in situ* as inhibitors of caspases and consequently programmed cell death [35].

To date, nothing is known about the expression of the other bovSERPINA3 members. In this study, we measured the expression levels of the eight *bovSERPINA3* genes using a quantitative real-time PCR based on TaqMan^®^ technology (custom assays), and we analysed bovSERPINA3 protein patterns in different tissues of 15-month-old Charolais bulls. For the first time, we demonstrate that all the bovSERPINA3 family members are expressed at transcriptional and translational levels. As previously described [28], we have defined two subgroups. Many studies have been performed on bovSERPINA3-1 and A3-3, two members of the first subgroup [32–35]. In this report, we focus on one member of the second subgroup, bovSERPINA3-7 that shows more difference in the RCL domain. We characterized bovSERPINA3-7 expression, its tissue distribution and its glycosylation. By immunoprecipitation, we revealed that bovSERPINA3-7 and creatine kinase M-type interact in skeletal muscle. By immunostaining, we showed that bovSERPINA3-7 is preferentially localized in fast-type fibres, precisely in the M-band sarcomere. We propose that bovSERPINA3-7 and creatine kinase type M could interact within these cells. These investigations of the subcellular and tissue distributions of bovSERPINA3-7 contribute to knowledge of the biological roles of serpins, especially in skeletal muscle cells. The workflow of this study is shown in the electronic supplementary material, figure S1.

## 2. Results

### 2.1. Transcriptional expression pattern of *bovSERPINA3* gene family

**Figure 1.**
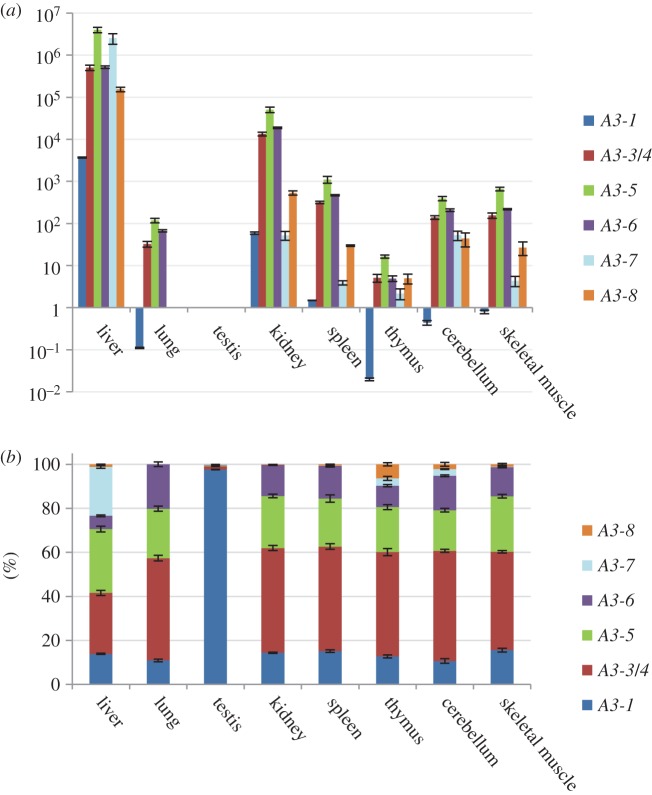
Tissue expression of *bovSERPINA3s* genes. (*a*) Relative amounts of transcripts (RQ ± S.D.) of *bovSERPINA3s* were determined by qPCR in 8 tissues. Each sample corresponds to 3 animals pooled cDNAs and was measured in triplicate. *bovSERPINA3s* expression levels were normalized by *TFIID*, one stable housekeeping gene and calibrated by testis tissue. The data were plotted on a logarithmic scale to simplify comparison of expression profiles. (*b*) Proportions of transcripts of *bovSERPINA3* genes for each tissue were calculated in per cent ± S.D.

The expression analysis of *bovSERPINA3* genes was carried out on eight different tissues from 15-month-old Charolais bulls (figure 1*a*). All *bovSERPINA3* genes are expressed at different levels in the different tested tissues. *BovSERPINA3* genes are highly expressed in the liver and weakly expressed in the lung, the testis and the thymus. In the other tissues (kidney, spleen, cerebellum and skeletal muscle), *bovSERPINA3* genes are expressed at intermediate levels. We also evaluated the contribution of each serpin expression in those tissues (figure 1*b*; electronic supplementary material, table S3), and we observed that the most related *bovSERPINA3* genes (*bovSERPINA3-1* to *A3-6*) are more expressed than the two most divergent members of the serpin family (*bovSERPINA3-7* and *A3-8*). At the transcriptional level, in each tested tissue except for liver and testis, *bovSERPINA3-3/4* and *bovSERPINA3-5* genes have nearly identical expression proportions. *BovSERPINA3-1* has the highest proportion of expression in testis compared with lung, thymus or cerebellum, and it is weakly expressed comparatively to other *bovSERPINA3* genes in liver, kidney, spleen and skeletal muscle. Surprisingly, *bovSERPINA3-6*, which belongs to the same subgroup, presents different expression proportions for each tissue except for kidney and spleen. *BovSERPINA3-8* has a similar expression proportion in liver, spleen, cerebellum and skeletal muscle and a different expression proportion in other tested tissues. Finally, the *bovSERPINA3-7* expression proportions are almost identical between testis and skeletal muscle and between thymus and cerebellum.

### 2.2. Expression patterns of bovSERPINA3 proteins in bovine tissues

**Figure 2.**
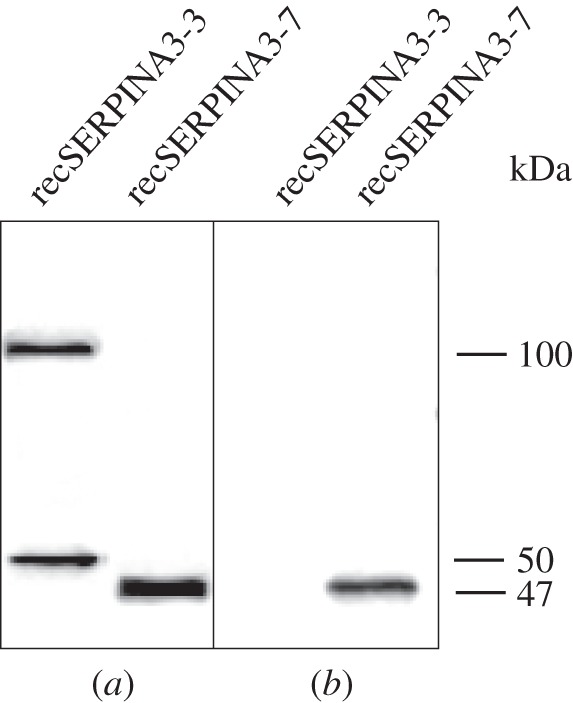
Specificities of polyclonal antibodies. The recombinant proteins bovSERPINA3-3 and A3-7 were separated on 12% reducing SDS-PAGE and analysed by Western blot using: (*a*) a polyclonal antibody raised against purified bovSERPINA3-1, or (*b*) a polyclonal antibody raised against a specific peptide of bovSERPINA3-7.

**Figure 3.**
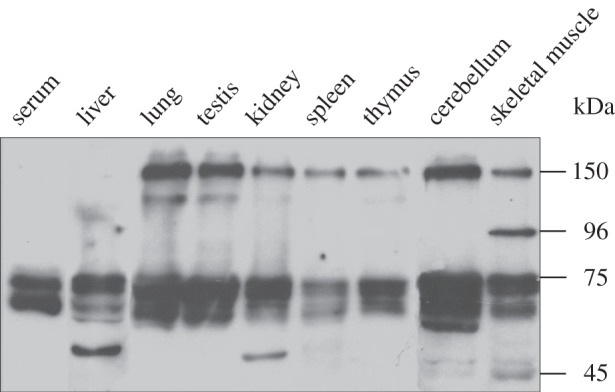
Protein expression of bovSERPINA3 family in a panel of bovine tissues. For each sample, total protein extract (40 µg) was prepared as described in §4.7, separated on 10% SDS-PAGE and analysed by Western blot using a polyclonal antibody raised against purified bovSERPINA3-1.

Although it is known that both bovSERPINA3-3 and A3-7 are expressed in skeletal muscles [32,33] and in chromaffin cells [30,31], nothing is known about the presence of the bovSERPINA3 family proteins in other tissues. To evaluate their distribution on diverse tissues of 15-month-old Charolais bulls, Western blot analyses were performed using a polyclonal antibody raised against purified bovSERPINA3-1. This antibody was evaluated for its capacity to recognize bovSERPINA3-3 and bovSERPINA3-7, two representative members of the bovSERPINA3 family. As previously described [36], on reducing SDS-PAGE, the antibody reveals the recombinant recSERPINA3-3 as a monomer form (50 kDa) and a dimeric form (100 kDa). This antibody also reveals the recombinant recSERPINA3-7 only under its monomeric form (47 kDa; figure 2*a*). Because this antibody is able to recognize one member of each subfamily, it may recognize the other bovSERPINA3 family members. For all tested tissues, the antibody reveals several bands in the range 45–150 kDa that could correspond to several (or all) bovSERPINA3 proteins with different post-translational modifications (figure 3). Moreover, these band patterns are quite different in each tissue. Except in the serum and the liver, a 150 kDa form is observed and could correspond to dimers. Indeed, some serpins such as bovSERPINA3-3 are known to dimerize under denaturing conditions and these dimers disappear on native PAGE [36,37]. Surprisingly, an additional band of 96 kDa is specifically observed in the skeletal muscle. We proposed that this 96 kDa band could be related to a muscle-specific conformation, a complex, or post-translational modifications of one of the most divergent serpins. To address these hypotheses, we designed a specific anti-bovSERPINA3-7 antibody allowing us to analyse specifically this protein.

### 2.3. Expression patterns of bovSERPINA3-7 protein in bovine tissues

We have produced both SERPINA3-3 [36] and A3-7 in *Escherichia coli*. We first evaluated the specificity of anti-bovSERPINA3-7 antibody against these bacterial proteins by Western blot. As shown in figure 2*b*, the antibody reacts with purified recSERPINA3-7, but not with recSERPINA3-3.

**Figure 4.**
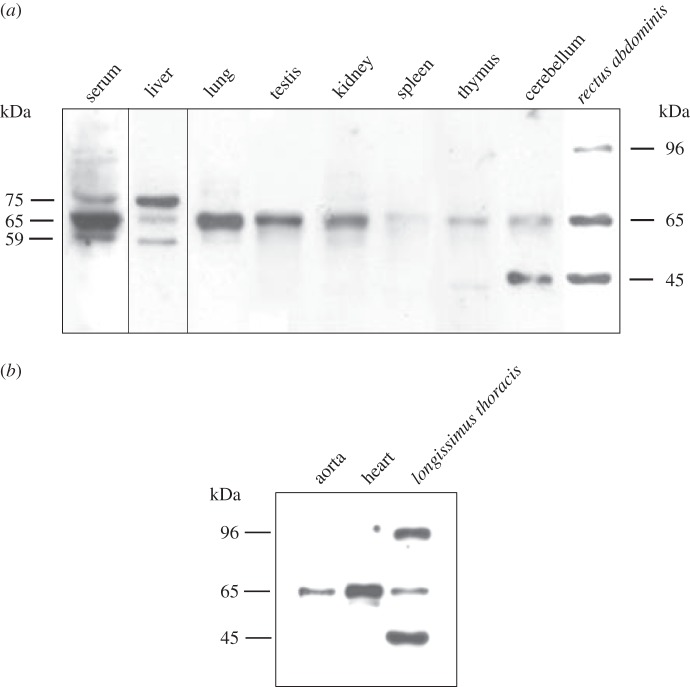
BovSERPINA3-7 protein expression. (*a*) In a panel of bovine tissues. (*b*) In a panel of bovine muscles. For each sample, total protein extract (40 µg) was prepared as described in §4.7, separated on 12% SDS-PAGE and analysed by Western blot using the polyclonal antibody raised specifically against bovSERPINA3-7.

Thus, we used this antibody to analyse the bovSERPINA3-7 patterns in several tissues of 15-month-old Charolais bulls by Western blot. For all samples, Western blot analysis reveals a protein at 65 kDa (figure 4*a*). This molecular weight is higher than that predicted from the amino acid sequence of bovSERPINA3-7 (44.17 kDa), i.e. without its signal peptide. Because four potential *N*-glycosylation sites are predicted in the protein sequence (UniProt data), the approximately 20 kDa difference in molecular weights between evaluated and apparent sizes of the protein could be explained by the presence of *N*-glycans at the four *N*-glycosylation sites. In addition to the 65 kDa band, we detected two other proteins of 59 and 75 kDa in the serum and the liver; these bands are not equally represented in these tissues, supporting the idea that bovSERPINA3-7 could be differentially glycosylated according to the tissue. In the cerebellum and skeletal muscle, an additional band of 45 kDa is observed and could correspond to the bovSERPINA3-7 without any post-translational modifications.

Finally, as shown in figure 4*a*, a band of 96 kDa is exclusively detected in skeletal muscle. To determine if this band is expressed in all the three muscle types, we also performed Western blot analyses on total proteins extracted from bovine *longissimus thoracis*, cardiac and smooth muscles (figure 4*b*). Surprisingly, whereas the 65 kDa band is observed in the three muscle types, the 96 kDa band is specifically expressed in skeletal muscle.

### 2.4. Glycosylation analyses of bovSERPINA3-7 in skeletal muscle

**Figure 5.**
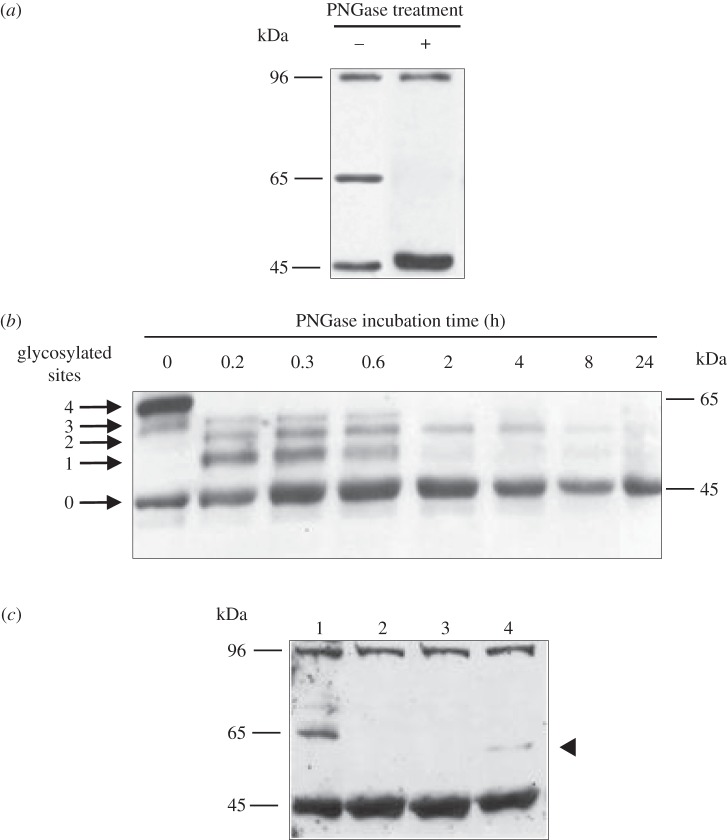
Glycosylation states of bovSERPINA3-7. (*a*) Bovine *longissimus thoracis* proteins (40 µg) were treated with PNGase F (2.5 units) for 16 h at 37°C. Proteins were revealed by Western blot using the polyclonal antibody raised against bovSERPINA3-7. (*b*) Kinetics of PNGase F digestion (2.5 units, 37°C) were performed on total proteins (40 µg) extracted from *longissimus thoracis* muscle. Five states of glycosylation (0–4) were detected by Western blot using the polyclonal antibody raised against bovSERPINA3-7. They correspond to fully, partially or non-glycosylated bovSERPINA3-7. (*c*) Deglycosylation assays of bovSERPINA3-7 were performed on proteins (50 µg) extracted from *longissimus thoracis* muscle. *Lane 1*: protein extract without treatment; *lane 2*: protein extract treated with PNGase F during 16 h; *lane 3*: protein extract treated with both PNGase F and a mixture of enzymes to remove *O*-glycans (sialidase A for cleavage of terminal sialic acid residues, *O*-glycosidase to remove the core Gal-β(1 → 3)-GalNAc, β(1 → 4)-galactosidase and β-*N*-acetylglucosaminidase to remove sugars associated with specific *O*-linked glycan structures) during 16 h; *lane 4*: protein extract treated with sialidase during 16 h.

UniProt data and our Western blot analyses suggested that bovSERPINA3-7 could be *N*-glycosylated at four putative sites. To confirm this hypothesis, we performed Western blot analysis on total proteins extracted from *longissimus thoracis* of 15-month-old Charolais bulls which were treated by different combinations of glycosidases. To remove *N*-glycans, we used the endoglycosidase PNGase F [38]. As indicated in figure 5*a*, PNGase F treatment of bovSERPINA3-7 induces the disappearance of the 65 kDa protein in favour of the 45 kDa which corresponds to the theoretical molecular weight of the non-glycosylated bovSERPINA3-7. To determine whether the four putative sites are *N*-glycosylated, partial digestion with PNGase F was performed on the same sample. As shown in figure 5*b*, bovSERPINA3-7 immunoblot revealed five different bands corresponding to the fully glycosylated protein at 65 kDa, the partially glycosylated protein at 60.5, 57.5 and 52 kDa, and the non-*N*-glycosylated protein at 45 kDa.

We completed our glycosylation analysis by submitting the muscle proteins to treatments allowing the removal of mucin-type *O*-glycans that are frequently present on secreted proteins [39]. The enzyme combinations to remove *N*- and *O*-glycans do not generate an additional shift of the molecular weight compared with the one obtained with the PNGase F alone (figure 5*c*). This result suggests that bovSERPINA3-7 in skeletal muscle is an *N*-glycoprotein harbouring four *N*-glycans and no type-mucin *O*-glycans. However, the treatment with the sialidase enzyme induces a shift in the migration of the serpin (form about 60 kDa indicated by arrow in figure 5*c*, lane 4). This result indicated that the *N*-glycans of bovSERPINA3-7 present in skeletal muscle are mainly sialylated.

Surprisingly, after 16 or 24 h of treatment, the 96 kDa band was still observed (figure 5*a,c*). Thus, this band does not reflect a muscle-specific glycosylation of bovSERPINA3-7. Moreover, as shown in figure 2, recSERPINA3-7 is not able to form a homodimer as is observed for recSERPINA3-3 in denaturing conditions. Altogether, these results indicate that the 96 kDa band is not related to a skeletal muscle-specific glycosylation of bovSERPINA3-7 or to a homodimer of non-glycosylated SERPINA3-7. Thus, we propose that this band could correspond to a skeletal muscle-specific complex of the non-glycosylated SERPINA3-7 with another/other protein partner(s).

### 2.5. Identification of a partner to bovSERPINA3-7 in skeletal muscle

**Figure 6.**
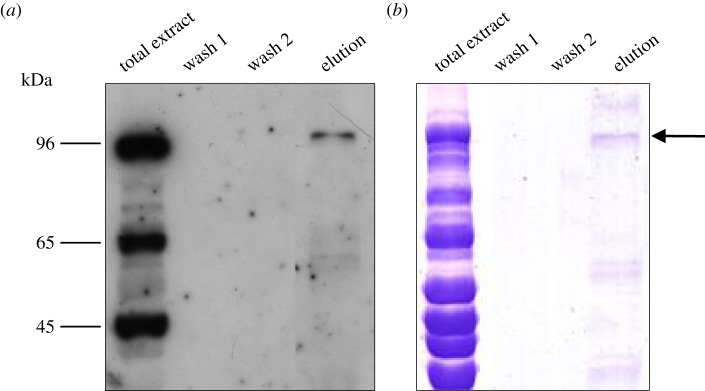
Immunoprecipitation assay of the 96 kDa complex. After immuoprecipitation, 10 µl of each wash fraction and elution fraction were analysed on 12% SDS-PAGE and the proteins revealed by (*a*) Western blot using the polyclonal antibody raised against bovSERPINA3-7 or (*b*) Coomassie blue staining. Total protein extract (40 µg) of *longissimus thoracis* was also analysed. The band of interest, indicated by an arrow, was excised from the gel and analysed by nanoLC-MS/MS as described in §4.14. The 65 kDa band corresponds to the mature glycosylated bovSERPINA3-7. The 45 kDa band corresponds to the non-glycosylated form of bovSERPINA3-7.

To identify the protein partner associated with bovSERPINA3-7 in *longissimus thoracis* of 15-month-old Charolais bulls, we performed an immunoprecipitation of bovSERPINA3-7 in this tissue. Western blot and Coomassie blue staining analyses revealed the presence of a 96 kDa band in the eluted fraction after bovSERPINA3-7 immunoprecipitation (figure 6).

Two samples of this 96 kDa band were analysed by nanoLC-MS/MS. The combined analysis for the two samples leads to the identification of bovine creatine kinase M-type with a coverage of 61% and a log(E) value of −68.77. Creatine kinase is the only protein detected with a high confidence level that has a molecular weight in accordance with the formation of a complex of 96 kDa with the bovSERPINA3-7 protein. Details of the analysis are given in electronic supplementary material, table S4.

The cytosolic creatine kinase M-type is an important player of muscle metabolism as it regulates the optimal ATP/ADP ratio during muscle contraction. As the bovSERPINA3-7 protein is expressed in skeletal muscle and could interact with creatine kinase M-type, we analysed the bovSERPINA3-7 localization in skeletal muscle by immunostaining.

**Figure 7.**
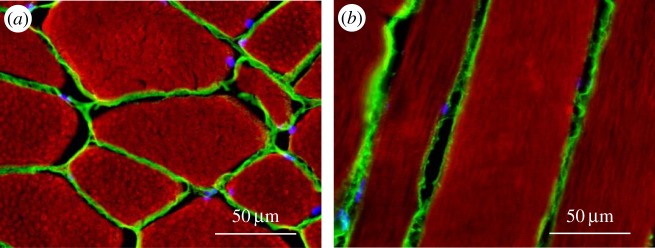
Immunolocalization of bovSERPINA3-7 in bovine skeletal muscle. Transversal (*a*) and longitudinal (*b*) 10 µm-thick sections of bovine *longissimus thoracis* were double immunostained with the polyclonal antibody raised against bovSERPINA3-7 and monoclonal antibody raised against laminin 2 alpha. Secondary specific fluorescent F(ab’)_2_ (Alexa Fluor^®^546 or Alexa Fluor^®^488, selectively linked to a goat anti-rabbit or a goat anti-rat F(ab’)_2_, respectively) were used for immunolocalization of each specific protein. Laminin (in green) localized to the membrane and bovSERPINA3-7 appears highly accumulated throughout the fibre.

Transversal and longitudinal sections of *longissimus thoracis* (figure 7*a* and *b*, respectively) showed that bovSERPINA3-7 is distributed throughout the individual muscle fibres. The co-immunostaining with an anti-laminin antibody confirmed that bovSERPINA3-7 is localized within the muscle fibres.

**Figure 8.**
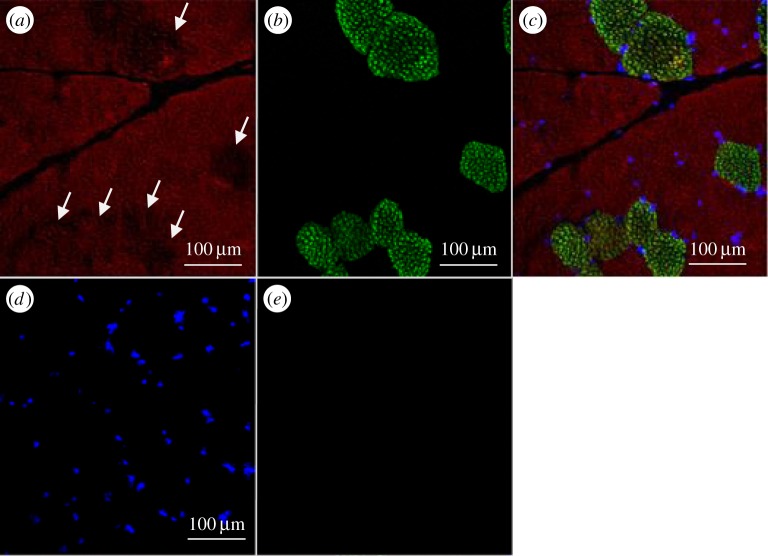
Immunolocalization of bovSERPINA3-7 in different fibre types of bovine skeletal muscle. Transversal 10 µm thick sections of bovine *longissimus thoracis* were double immunostained using the polyclonal antibody raised against bovSERPINA3-7 (*a*) and a monoclonal anti-slow skeletal myosin heavy chain antibody (*b*). Secondary specific fluorescent F(ab’)_2_ (Alexa Fluor^®^546 or Alexa Fluor^®^488, selectively linked to a goat anti-rabbit or a goat anti-mouse F(ab’)_2_, respectively) were used for immunolocalization of each specific protein. The merged image is shown in (*c*). In the transverse sections, nuclei were stained with DAPI (*d*). Control for immunofluorescence staining was performed in the same conditions using the pre-immunoserum of bovSERPINA3-7 (*e*). Type II fibres have a higher accumulation of bovSERPINA3-7 compared with type I fibres (arrows).

The immunostaining also revealed that all the fibres are not equally stained (figure 8*a*). Muscle fibre types were then characterized according to their myosin isoforms. We observed that the bovSERPINA3-7 protein is less expressed in type I compared with type II fibres (figure 8*c*). According to these results, we concluded that the bovSERPINA3-7 protein is mainly expressed in fast-type (type II) fibres and partially in slow-type (type I) fibres.

**Figure 9.**
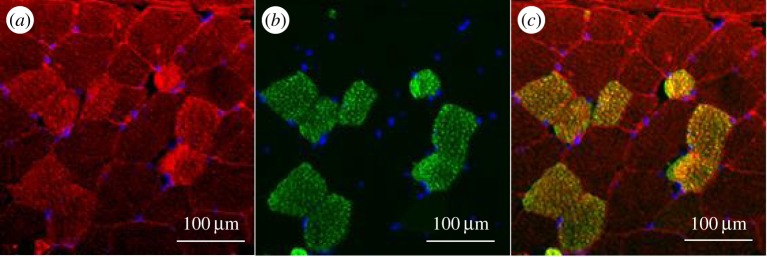
Immunolocalization of bovSERPINA3 family in different fibre types of bovine skeletal muscle. Transversal 10 µm thick sections of adult bovine *longissimus thoracis* were double immunostained using the polyclonal antibody raised against bovSERPINA3 family (*a*) and a monoclonal anti-slow skeletal myosin heavy chain antibody (*b*). Secondary specific fluorescent F(ab’)_2_ (Alexa Fluor^®^546 or Alexa Fluor^®^488, selectively linked to a goat anti-rabbit or a goat anti-mouse F(ab’)_2_, respectively) were used for immunolocalization of each specific protein. The merged image clearly indicated that all type I fibres of adult bovine *longissimus thoracis* muscle accumulate high levels of bovSERPINA3s (*c*). In contrast, type II fibres have lower levels of bovSERPINA3s proteins compared with the type I fibres.

**Figure 10.**
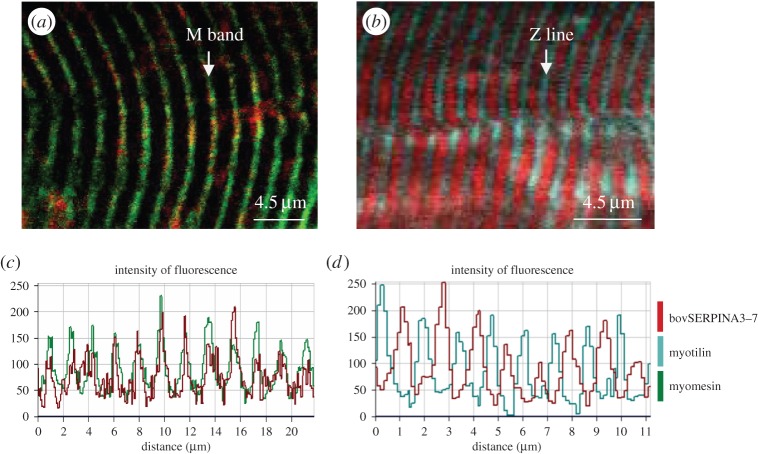
Immunolocalization of bovSERPINA3-7 in bovine skeletal myofibre sarcomere. Longitudinal 10 µm thick sections of bovine *longissimus thoracis* were analysed by confocal microscopy. Double staining was performed using the polyclonal antibody raised against bovSERPINA3-7 and polyclonal anti-myomesin-I (*a*). Secondary specific fluorescent F(ab’)_2_ (Dylight^®^594 or Dylight^®^488, selectively linked to a donkey anti-rabbit or a donkey anti-goat F(ab’)_2_, respectively) were used for immunolocalization of each specific protein. Double staining was performed using the polyclonal antibody raised against bovSERPINA3-7 and monoclonal anti-myotilin (*b*). Secondary specific fluorescent F(ab’)_2_ (Alexa Fluor^®^546 or Alexa Fluor^®^488, selectively linked to a goat anti-rabbit or a goat anti-mouse F(ab’)_2_, respectively) was used for immunolocalization of each specific protein. BovSERPINA3-7 clearly localizes to the sarcomeric M-band as shown by the colocalization with myomesin-I (yellow in *a*). In addition, the alternating signal of myotilin (Z-disc component) and bovSERPINA3-7 (*b*) confirms this result, which is further illustrated by the RGB profile seen in (*c*) and (*d*).

Analysis by confocal microscopy, using the antibody raised against the bovSERPINA3 family, showed that all the fibres are stained (figure 9*a*), and the intensity of fluorescence is variable according to fibre type. Contrary to our observation for bovSERPINA3-7, the double staining SERPINA3s/type I myosin reveals that most of the bovSERPINA3 family proteins are preferentially expressed in type I fibres (figure 9*c*). Therefore, in skeletal muscle, it seemed that the bovSERPINA3-7 protein is preferentially expressed in type II fibres, whereas the other members of the bovSERPINA3 family (or most of them) are expressed in type I fibres. Finally, the longitudinal sections of bovine *longissimus thoracis* muscle allowed us to localize bovSERPINA3-7 inside the sarcomeric structures including the Z-disk and M line identified by myotilin and myomesin stainings, respectively. As shown in figure 10, confocal imaging indicates clearly that bovSERPINA3-7 localizes into the sarcomeric M-band as established by its colocalization with myomesin (yellow in figure 10*a*). The RGB profile along a line (figure 10*c*) confirms the colocalization of these two proteins and clearly demonstrates that bovSERPINA3-7 is localized between two Z-discs stained by myotilin (figure 10*b,d*). Taken together, these data revealed that within skeletal muscle fibres, bovSERPINA3-7 is specifically localized in the sarcomeric M-band where it could interact with other specific M-band component(s).

## 3. Discussion

In contrast to human, where the serpin A3 is represented by a unique gene and a unique protein α1-ACT, the situation seems to be more complex in cattle and remains original in mammals. The family of bovine SERPINA3s contains a subgroup of six closely related proteins bovSERPINA3-1 to bovSERPINA3-6 and two other members, bovSERPINA3-7 and bovSERPINA3-8, that present differences, notably in the RCL [28]. This suggests that these proteins have different physiological roles.

To gain knowledge on the potential physiological role(s) of each serpin, it is essential to examine their expression in various tissues. For that purpose, studies at both transcriptional and translational levels were carried out on all bovSERPINA3s. In this study, we were able to quantify at the transcriptional level the expression of each gene. Our results confirmed that bovSERPINA3 family members are ubiquitously expressed in all Charolais-tested tissues. Except in the liver (major secretory organ of SERPINA3) which presents a high level of each transcript, their expressions are highly different for other tissues. Thus, for the same tissue, the mRNA expression level can be very different between *bovSERPINA3* genes. In the same manner, for the same *bovSERPINA3* gene, its expression level varies between tissues. Whereas *bovSERPINA3* genes are located on the same locus and/or cluster, these results suggest different mechanisms of regulation for the expression of each member of the bovine family.

Because of the very high sequence similarity between the eight proteins, it is difficult to produce a specific antibody for each serpin. However, using a polyclonal antibody, we detected all these serpins in a panel of bovine tissues. Although bovSERPINA3 family members are ubiquitously expressed, their translational expression levels are highest in the liver and serum. This result is in agreement with the liver being the major secretory organ of several serpins, including α_1_-ACT and α_1_-antitrypsin (AAT) as described in humans [18]. Moreover, these serpins represent the most abundant serpins in human plasma. These potent plasma inhibitors are capable of inhibiting several serine proteases involved in inflammation [40] or leucocyte-derived proteases that are released at the site of skin injury [41].

For this original bovine family, it was essential to determine more specifically which SERPINA3s are present in these bovine-tested tissues. The present evidence that mRNAs and proteins of some of these serpins are expressed in different tested tissues clearly indicates a complexity of the potential physiological role(s) of these inhibitors. Using the antibody which recognized all members of bovSERPINA3 family, the range of molecular weight detected by Western blot results from the expression of several members of this family in the same tissue and/or the presence of more than one glycoform of each serpin. Indeed, in addition to the bands of about 45 kDa (which correspond to the non-glycosylated forms of bovSERPINA3s) observed in the liver, kidney and skeletal muscle, several other bands corresponding to different glycosylated states were also detected in all tested tissues. This particular status has been described for the protein C inhibitor (PCI or SERPINA5) which is a serpin type of serine protease inhibitor. The *N*-glycosylation of the seminal plasma PCI differs strikingly compared with those of both blood-derived and urinary PCI. In addition, the PCI glycoforms displayed different activities. The authors concluded that the *N*-glycans of PCI are tissue-specific and could be responsible for PCI conformational changes [42]. Similarly, glycosylation variants of human corticosteroid-binding globulin (CBG or SERPINA6) have been characterized. Importantly, some of them have been shown to affect the affinity of CBG for its receptors [43–45].

One intriguing question in the field remains regarding the biological significance of the eight closely related SERPINA3 proteins in cattle. In this study, we focused on the characterization of bovSERPINA3-7, one of the two most different members of the bovSERPINA3 family. We took advantage of a sequence upstream of the bovSERPINA3-7 RCL, which differs from the one found in the other serpins, to design a specific anti-bovSERPINA3-7 antibody. The expression pattern of this protein illustrates perfectly the complexity previously described. In all tested tissues, bovSERPINA3-7 is expressed as an *N*-glycosylated protein of 65 kDa. The 59 kDa band, observed in both serum and liver, corresponds to bovSERPINA3-7 with an intermediate glycosylated state, as it disappears after PNGase F treatment (data not shown). A form without post-translational modification (45 kDa) is also detected in cerebellum and muscle. For the glycosylated forms, total and partial deglycosylation experiments proved that bovSERPINA3-7 is a glycoprotein with sialylated *N*-glycans. To complete this study and that of Hwang *et al*. [31], we suggest that the four sites of bovSERPINA3-7 are *N*-glycosylated. Surprisingly, both in liver and serum, the 75 kDa band revealed by the polyclonal antibody specific for bovSERPINA3-7 is not affected by PNGase F treatment (data not shown) and remains to be identified. Furthermore, a 96 kDa band, resistant to PNGase, is detected only in skeletal muscles of 15-month-old Charolais bulls with both antibodies raised against bovSERPINA3-7 and all bovSERPINA3s. Our results indicate that bovSERPINA3-7 does not exist in a homodimeric form unlike bovSERPINA3-3 (see §2.2). In previous data [34], we have shown that bovSERPINA3-3 expressed in *E. coli* (without *N*-glycans) or purified from muscle (*N*-glycosylated) remains active and displays comparable association rate constants when tested against trypsin and caspase 3. To assert that the recombinant protein bovSERPINA3-7 corresponds to an active serpin, the *in vitro* inhibitory activity was tested against elastase. We found that recSERPINA3-7 inhibits this serine protease with an association rate (*k*_ass_) of 1.7 × 10^5^ M^−1^ s^−1^, a similar value to that measured by Hwang *et al*. [31]. This suggests that non-glycosylated bovSERPINA3-7 is functional *in vivo*. Therefore, we can assume that the 96 kDa band correspond to a stable heterodimer of non-glycosylated bovSERPINA3-7 (45 kDa) and a protein partner (about 50 kDa).

NanoLC–MS/MS analysis of the 96 kDa-specific skeletal muscle band reveals the presence of bovine creatine kinase M-type. Muscle cytosolic creatine kinase isoform plays an important role in muscle energetic metabolism and is a very efficient system of maintaining optimal ATP/ADP ratio during muscle contraction. The active form of this enzyme is a homodimer of 80–86 kDa [46]. As reported, creatine kinase migrates at 43 kDa under denaturing conditions [47]. The association between this monomeric form and a non-glycosylated form of bovSERPINA3-7 can achieve a complex of 96 kDa, as observed. In addition, it is well documented that creatine kinase M-type is located in the M-band of the sarcomere [48] where it interacts with myomesin and M protein [49], especially in fast-twitch skeletal muscles [50]. More recently, computational simulations to predict creatine kinase-associated factors [51] have shown that creatine kinase M-type can potentially interact with 85 significant partners. Among these proteins, there are M-band components such as myomesin, M-band protein, titin and myosin. The function of most of the other candidate proteins has not been reported yet and remains unknown in skeletal muscles. Among them, we note the presence of SERPINF1, a strong inhibitor of angiogenesis [52]. SERPINF1 presents a full-length significant homology of about 50% with bovSERPINA3-7. Using immunostaining, we showed in this work that bovSERPINA3-7 is preferentially present within the sarcomeric M-band of type II (fast-type) muscle fibres and co-localizes with myomesin, supporting the hypothesis of an interaction between bovSERPINA3-7 and creatine kinase M-type in skeletal muscle. While most of the other bovSERPINA3s are detected in type I (slow-type) fibres, A3-7 is preferentially observed in type II fibres, suggesting that bovSERPINA3-7 plays a specific role in skeletal muscle. The determination of partners can further promote our understanding of the physiological role(s) of bovSERPINA3-7.

## 4. Material and methods

### 4.1. Samples

For quantitative real-time PCR and Western blot analyses, tissue samples were obtained from three 15-month-old Charolais bulls. All tissue samples were collected from young entire males of pure breed Charolais: animals (12 months old at start) were assigned to a 100 day finishing period before slaughter. They were housed in groups in 6 × 6 m pens with straw bedding and individually fed and weighed every two weeks. Diets consisted of concentrate (75%) and straw (25%). Animals were slaughtered at the same age (15 months). The tissues collected included liver, spleen, thymus, cerebellum, kidney, lung, testis, serum, skeletal muscles (*longissimus thoracis* and *rectus abdominis*), aorta and heart. All these bovine tissues were obtained from the INRA experimental slaughterhouse (Theix, France). The specimens used for all experiments were snap frozen upon extraction and stored at −80°C until use.

### 4.2. Frozen sections

Skeletal muscle *longissimus thoracis* from 15-month-old Charolais bulls was dissected, snap frozen in isopentane, cooled by liquid nitrogen and stored at −80°C until sectioning. Cryosections of samples were cut using a Reichert Frigocut 2800 (Leica, Heidelberg, Germany) with a cutting temperature of about −20°C. Thick sections of 10 µm were made across or longitudinally to the muscle fibres. Cryosections from each tissue sample were collected on Teflon printed diagnostic (Immuno-Cell, Mechelen, Belgium) and dried for 1 h at room temperature before treatments.

### 4.3. RNA extraction and first strand cDNA synthesis

Total RNA was isolated from tissue samples using the RNeasy Midi kit (Qiagen, Hilden, Germany) according to the manufacturer's instructions. The digestion with DNAse I (Qiagen) was performed on the spin column. Concentration of the isolated RNA and the 260/280 nm absorbance ratio were measured with the NanoDrop^®^ ND-1000 spectrophotometer (Thermo Scientific, Wilmington, DE). RNA integrity was additionally assessed using the Agilent 2100 Bioanalyser (Agilent Technologies, Santa Clara, CA). All samples had RIN ≥ 8. RNA was stored at −80°C when the reverse transcription step was not immediate. Two micrograms of total RNA were reverse transcribed in a total volume of 20 µl using 250 ng of random hexamers primers (Invitrogen, Carlsbad, CA) and 200 units of Superscript™ III Reverse Transcriptase (Invitrogen) according to the manufacturer's guidelines. The reaction was incubated for 5 min at 25°C, 60 min at 50°C and enzyme was inactivated for 15 min at 70°C. cDNAs were stored at −20°C until use.

### 4.4. Primers and probes design

Each set of primers and the corresponding probe were chosen to be as specific as possible for the different transcripts. Primers and TaqMan^®^ probes were designed using the primer analysis software Primer Express v. 2.0 (Applied Biosystems, Foster City, CA). Primers were synthesized by MWG Biotech (Courtaboeuf, France) and TaqMan^®^ FAM dye-labelled probes by Applied Biosystems.

### 4.5. Quantitative real-time PCR

The sets of primers (table 3) were selected for their reaction efficiencies (close to 100%) determined from calibration curves and melting curve analysis. These primers were validated with cDNA dilution series (10^−1^, 10^−2^, 10^−3^, 10^−4^ and 10^−5^) from adult liver cDNA. For each set of primers, melting curve analyses were done and PCR products were purified and sequenced for confirmation. Each assay was performed with three pooled cDNA samples and was made in triplicate. No acceptable suitable set of specific primers was found for *bovSERPINA3-2*. Only a common set of primers was designed for *bovSERPINA3-3* and *bovSERPINA3-4*. Transcription factor IID (*TFIID*) was used as internal RNA control to normalize samples for expression.

**Table 3.** Primers sequences for qPCR and accession numbers of genes.

primer and probe names	nucleotide sequences	transcript targets	accession numbers
SA3-1F SA3-1R TMSA3-1	5′-CGACAGCGCCCTCTTCATC-3′ 5′-GAGACTACCAGGTCCGCGGT-3′ 5′-AGCCTCCAGCAACACTGACTTCGCC-3′	*bovSERPINA3-1*	GenBank: AY911536
SA3-3/4F SA3-3/4R TMSA3-3/4	5′-CATGAGGGCAGAGAGACTGTCC-3′ 5′-CTTCTGTGCCGGTCCTTCAC-3′ 5′-CCCCTCCTGGCTCTGGGGCTC-3′	*bovSERPINA3-3* and *A3-4*	GenBank: AY911537 GenBank: EF153627
SA3-5F SA3-5R TMSA3-5	5′-CTTCCCGACCAACTTCAGGG-3′ 5′-GTCATCATGGGCACCTCCAC-3′ 5′-TGGTGAAGGACCAGCGCAGAAGG-3′	*bovSERPINA3-5*	GenBank: EF153628
SA3-6F SA3-6R TMSA3-6	5′-ACCCCTTACTTCCGGGACG-3′ 5′-ACAGCAGCTCCTTCCGTGC-3′ 5′-CACTGCCTCCCAGAATGTGACCCCA-3′	*bovSERPINA3-6*	GenBank: EF153629
SA3-7F SA3-7R TMSA3-7	5′-AGCGCCCTCTTCATCCTCC-3′ 5′-TCCTTCCGTGCCCTCCTC-3′ 5′-ACCACAAGCTGGCAGTTTCCCACG-3′	*bovSERPINA3-7*	GenBank: EF153630
SA3-8F SA3-8R TMSA3-8	5′-CTGGGGCTCCTGGTGTCTG-3′ 5′-GGAAGGCCAAGGCTATGGAG-3′ 5′-CAAGAACAAGACGGTGGAGGTGCCC-3′	*bovSERPINA3-8*	GenBank: EF153631
TFIID-F TFIID-R TMTFIID	5′-CGTGCCCGAAATGCTGAATA-3′ 5′-TTCACTCTTGGCTCCTGTGCA-3′ 5′-TAAGAGAGCCCCGCACCACTGCA-3′	*TFIID*	GenBank: NM_001075742

Using optimized qPCR conditions, all targeted mRNAs were detected using TaqMan^®^ technology in all examined bovine tissues. For each set of experiments, the results were calibrated using the value of the testis expressed genes. The cycle threshold (*C*_t_) values varied (electronic supplementary material, table S1) indicating that transcript abundance is gene and tissue-related.

Quantitative real-time PCR was carried out in triplicate using the ABI PRISM^®^ 7900HT Sequence Detection System (Applied Biosystems) in 96 well microtitration plates. Each qPCR was performed in a final volume of 20 µl containing 8 µl of appropriate dilution cDNA template (three pooled equivalent samples), 10 µl of MasterMix (2× TaqMan™ Universal PCR MasterMix, no amperase UNG-Applied Biosystems), 200 nM of labelled probe and 300 nM of forward and reverse primers. The qPCR protocol was as following: denaturation by a hot start at 95°C for 10 min followed by 40 cycles of a two-step programme (denaturation at 95°C during 15 s and annealing/extension at 60°C for 1 min). qPCR data were analysed using the appropriate threshold set-up as recommended by Applied Biosystems (SDS 2.3) before being transferred to Microsoft Excel. The slope of the calibration curve was calculated from the plot of log_10_ of initial target copy number versus the corresponding *C*_t_. The PCR efficiency (*E*) was determined from the slope of the curve obtained with serially diluted samples, as *E* = 10^(–1/slope)^. In a sample, the expression levels of target genes are normalized to the endogenous control (average of 2). This is given by Δ*C*_t_, were Δ*C*_t_ is determined by subtracting the average endogenous gene *C*_t_ value from the average target gene *C*_t_ value [*C*_t_ target gene–*C*_t_ average (endogenous gene)]. The calculation of ΔΔ*C*_t_ involves subtraction of the Δ*C*_t_ value for the controls from the Δ*C*_t_ value for the cases [Δ*C*_t_ target gene_(case)_ − Δ*C*_t_ target gene_(control)_]. The RQ value (RQ = 2^−ΔΔ*C*_t_^) is the relative expression of the target gene compared with the control (electronic supplementary material, table S2). Proportions of transcripts of *bovSERPINA3* genes are calculated by subtraction of *C*_t_ target gene_(case)_ from the number of cycles divided by the addition of [*C*_t_ target gene_(case)_ − number of cycles] and are expressed as percentages (electronic supplementary material, table S3). Data were transferred to Microsoft Excel, and proportions of transcripts of *bovSERPINA3* genes were statistically evaluated through numerous pairwise comparisons using Student's *t*-test. Differences were considered significant when *p* < 0.05.

### 4.6. Expression and purification of recombinant bovSERPINA3-7

Expression plasmid encoding mature bovSERPINA3-7, excluding the putative signal peptide, was constructed in pET19b plasmid vector (Novagen, Madison, WI) as previously described for bovSERPINA3-3 [36]. The bovSERPINA3-7 protein was expressed in *Escherichia coli* BL21-RP CodonPlus (DE3)-RP strain (Stratagene, La Jolla, CA). Protein was expressed with an NH_2_–His tag to the N-terminus, which allows affinity purification on a Ni^2+^ column. Purification was performed using a Ni-NTA Fast Start column (Qiagen) according to the manufacturer's instructions, and subsequent dialysis.

### 4.7. Protein extraction and SDS-PAGE analyses

One hundred milligrams of tissue pieces and 800 µl of extraction buffer (Tris-HCl 50 mM pH 7.5, KCl 150 mM, EDTA 4 mM) supplemented with protease inhibitor cocktail tablets (Roche Diagnostics, Mannheim, Germany) and 200 mM β-mercaptoethanol were added to a 2 ml lysis Matrix E tube (MP Biomedicals, Santa Ana, CA). The tube was processed in the FastPrep FP120 instrument (Thermo Savant, Holbrook, NY) for three periods of 20 s at a setting of 6. After homogenization, the tube was centrifuged at 10 000*g* for 1 min at 4°C and supernatant was collected. Concentrations of the proteins extracted from bovine tissues were measured as described in [53]. SDS-PAGE was performed as described previously [54], under reducing conditions on 10% or 12% acrylamide separating gels. Proteins (40 µg) were solubilized with 2× reducing loading buffer (2% SDS, 20% glycerol, 100 mM Tris-HCl, pH 6.8, 0.1% bromophenol blue, 5% β-mercaptoethanol). Molecular masses were estimated using the Precision Plus Protein Standards calibration kit (BioRad, Hercules, CA). Proteins were revealed with 0.25% Coomassie brilliant R-250 solution.

### 4.8. Antibodies

Six primary antibodies were used in this study. Polyclonal anti-bovine SERPINA3s antibody was raised against purified bovSERPINA3-1 [55]. It cross-reacted with all of the bovine SERPINA3 proteins. Rabbit antiserum specific for bovSERPINA3-7 (Agro-Bio, La Ferté St. Aubin, France) was raised in rabbit against a synthetic peptide FFKAQWKTPFNPNHTYES found only in the amino acid sequence of bovSERPINA3-7 and in no other animal or bacterial protein. Immunoglobulins (IgGs) from antisera were purified by protein A sepharose according to the manufacturer's protocol (GE Healthcare, Chalfont St Giles, UK) and used for immunoblotting at a dilution of 1 : 1500.

Four commercial antibodies were also used in this study: IgG1 mouse monoclonal anti-myotilin (A. Menarini Diagnostics France Bond & Novocastra Reagents, Rungis, France), goat polyclonal anti-myomesin-1 (Santa Cruz Biotechnology, Information Systems, Santa Cruz, CA), IgG1 mouse monoclonal anti-slow skeletal myosin heavy chain antibody (Abcam, Paris, France) and rat monoclonal anti-laminin 2 alpha (Abcam).

For Western blot analyses, a second antibody, swine anti-rabbit IgG conjugated to horseradish peroxidase (DAKO, Glostrup, Denmark), was used (dilution 1 : 1000). For microscopy analyses, several second antibodies were used and are described in §4.16.

### 4.9. Western blot analyses

Separated proteins were then transferred onto a PVDF Western blotting membrane (Roche Diagnostics) and electroblotted for 40 min at 200 mA. After overnight saturation at 4°C, the membrane was first incubated under agitation with a primary antibody for 1 h at 20°C, and then with a second antibody conjugated to horseradish peroxidase. The immunoblot was processed by chemiluminescence detection (Chemiluminescence Blotting Substrate (POD), Roche Molecular Biochemicals, Mannheim, Germany).

### 4.10. Deglycosylation

For deglycosylation assays, protein extracts were treated with different combinations of enzymes from an enzymatic protein deglycosylation kit (Sigma-Aldrich, St Louis, MO) according to the manufacturer's instructions. *O*-Deglycosylation assays required the use of sialidase A for cleavage of terminal sialic acid residues, *O*-glycosidase to remove the core Galβ(1 → 3)-GalNAc, β(1 → 4)-galactosidase and β-*N*-acetylglucosaminidase to remove sugars associated with specific *O*-linked glycan structures. To remove all *N*-linked glycans, we used the PNGase F.

### 4.11. Enzyme assay

Porcine pancreatic elastase (Calbiochem, La Jolla, CA) was titrated using human plasmatic α_1_-antitrypsin (Calbiochem). The activity was determined by using the fluorogenic substrate MeOSuc-AAPV-AMC (Calbiochem). Equimolar amounts of active enzyme and bovSERPINA3-7 (10 nM) were used for kinetic studies to determine the association rate constant (*k*_ass_). The serpin was pre-incubated with elastase in 50 mM Tris-HCl buffer, pH 8.0, containing 10 mM CaCl_2_ at room temperature (25°C) for given periods of time. Substrate (100 µM) was then added, and residual enzyme activity was measured at timed intervals. The second-order rate constant *k*_ass_ was computed as the slope of the plot of the reciprocal of free enzyme 1/[*E*] over time of incubation with inhibitor (*t*), based on the kinetic equation as 1/[*E*] = *k*_ass_ (*t* + 1/[*E*_o_], where [*E*_o_] = initial enzyme concentration and [*E*] = residual enzyme concentration [56].

### 4.12. Immunoprecipitation

Anti-bovSERPINA3-7 polyclonal antibody was used for immunoprecipitation experiments. In first step, IgG antibodies were purified from rabbit serum on a Hitrap protein AHP column packed with 1 ml of protein A sepharose (GE Healthcare Bio-Science, Uppsala, Sweden) according to the manufacturer's instructions. Purified immunoglobulins were dialysed and then chemically cross-linked with the protein AG, a genetically engineered protein which combines the IgG binding domains of both proteins A and G, coated on the surface of the magnetic beads. Dissected muscle from *longissimus thoracis* (20 mg) was homogenized and solubilized in extraction buffer (Tris-HCl 50 mM, pH 7.5, KCl 150 mM, EDTA 4 mM, pH 8.0). After centrifugation at 10 000*g* for 20 min at 4°C, the resulting supernatant was incubated with 20 µl of beads for 30 min at room temperature under agitation. After four washing steps with an excess of neutral extraction buffer, immunocomplex was collected with 50 µl of acid elution buffer (glycine 0.1 M, SDS 0.1%, pH 2.5) and immediately neutralized with 3 µl Tris-HCl 1 M, pH 9.0. Aliquots were analysed by Western blot using the specific antibody relative to the component that we intended to detect.

### 4.13. In-gel protein digestion

All chemical products for mass spectrometry analysis were purchased from Sigma (St Louis, MO). Sequencing grade-modified trypsin used for protein digestion was purchased from Promega (Charbonnières, France). Bands of interest were excised from the gel and in-gel digestion was performed as described [57] with minor modifications. Briefly, bands were destained and vacuum dried. After washes (acetonitrile 50% (twofold), NH_4_HCO_3_ 25 mM, acetonitrile 50%, acetonitrile 100%), proteins were reduced with 25 mM DTT in 100 mM NH_4_HCO_3_ for 60 min at 56°C, and alkylated with 25 mM iodoacetamide in 100 mM NH_4_HCO_3_ for 60 min at room temperature in the dark. Trypsin digestion was performed overnight at 37°C (20 ng µl^−1^ in 25 mM NH_4_HCO_3_). The resulting peptides were extracted successively by acetonitrile 50%–formic acid 0.25% (twofold), acetonitrile 100%. Extracted fractions were pooled and vacuum dried overnight and resuspended in 25 µl of acetonitrile 2%–trifluoroacetic acid 0.08% for mass spectrometry analysis.

### 4.14. LTQ-Orbitrap analysis and database searching

Four microlitres of the tryptic digest was analysed by nanoLC-MS/MS using LTQ-Orbitrap Discovery mass spectrometer (Thermo Scientific) interfaced with an Ultimate^™^ 3000 RSLCnano System (Thermo Scientific). Peptides were separated on a 2 µm C_18_ PepMap column (Thermo Scientific). Mass data collected during analysis were processed by the open source Xtandem pipeline parser 3.3 (The Global Proteome Machine, http://www.thegpm.org) and the MS/MS data were used to query databases Uniprot_Bostaurus_18072012.fasta, \contaminants_standards.fasta and Oryctolagus_cuniculus.fasta with the following criteria: 0.5 Da for peptide and fragment mass tolerances, one missed trypsin cleavage site allowed, carbamidomethylation of cysteine residues (from iodoacetamide exposure) and methionine oxidation as variable modifications. The protein identification, filtered for bovine and rabbit species, was established for protein score: logEvalue prot. <−2.6, peptide score: Evalue pep. <0.05 at least two peptides, and automatic elimination of the result set on the contaminating proteins.

### 4.15. Immunostaining

Cryosections were first blocked in phosphate-buffered saline (PBS) with 10% goat serum for 1 h at room temperature. After two washing steps of 3 min with PBS, primary antibodies were added (range of dilution: 1/400–1/20 according to the used antibody) and incubated for 1 h at room temperature. After two other washing steps of 3 min, secondary antibodies were added (range of dilution: 1/400–1/200 according to the used antibody) for 50 min at room temperature. Samples were washed again two times in PBS for 3 min before adding DAPI (dilution: 1/1000; Invitrogen-Life Technologies, Saint Aubin, France). The specimens were finally washed for a last time for 3 min and mounted on glass slides with Fluoromount™ Aqueous Mounting Medium (Sigma-Aldrich, Saint-Quentin Fallavier, France).

Controls for immunofluorescence staining were treated as above under the same conditions, except that the primary antibody was replaced by pre-immune serum or the same class of immunoglobulin (for an isotypic control). A final control of the specificity of the immunochemical procedure was checked by incubation of cryosections with the secondary antibodies without primary antibodies.

All incubations were carried out in a humid atmosphere.

### 4.16. Immunofluorescence light microscopy

Immunoreactions were detected with indirect fluorescence using F(ab’)_2_ goat IgG anti-mouse Alexa Fluor^®^488 (Invitrogen-Life Technologies) or F(ab′)_2_ donkey IgG anti-goat Dylight^®^488 (Abcam) or goat IgG anti-rat Alexa Fluor^®^488 (Invitrogen-Life Technologies) for green fluorescence and F(ab′)_2_ goat IgG anti-rabbit Alexa Fluor^®^546 (Invitrogen-Life Technologies) or F(ab′)_2_ donkey IgG anti-rabbit Dylight^®^594 (Abcam) for red fluorescence.

Double labelling was performed simultaneously using one monoclonal and one polyclonal antibody followed by two secondary antibodies coupled to fluorochromes of different wavelengths.

Microscopy analyses were carried out using an epifluorescence Leica Digital Microscope inverted DMI 6000B (Leica Microsystèmes SAS All Microscopy and Histology, Nanterre, France). Image processing was done using the image processing software MetaMorph. Confocal laser microscopy analyses were performed using a laser scanning microscope Zeiss LSM 510 META (Carl Zeiss Jena GmbH, Jena, Germany). Images were recorded and processed using Volocity^®^ three-dimensional image analysis software (PerkinElmer, San Jose, CA).

## Supplementary Material

Table S1. Table S2. Table S3. Table S4. Figure S1

## References

[1] Irving JA, Pike RN, Lesk AM, Whisstock JC 2000 Phylogeny of the serpin superfamily: implications of patterns of amino acid conservation for structure and function. Genome *Res* 10, 1845–1864. (doi:10.1101/gr.147800) 10.1101/gr.gr-1478r 11116082

[2] Rawlings ND, Tolle DP, Barrett AJ 2004 Evolutionary families of peptidase inhibitors. Biochem. J. 378, 705–716. (doi:10.1042/BJ20031825) 14705960 10.1042/BJ20031825 PMC1224039

[3] Olson ST, Gettins PG 2011 Regulation of proteases by protein inhibitors of the serpin superfamily. Prog. Mol. Biol. Transl. Sci. 99, 185–240. (doi:10.1016/B978-0-12-385504-6.00005-1) 21238937 10.1016/B978-0-12-385504-6.00005-1

[4] Patterson SD 1991 Mammalian *α*_1_-antichymotrypsins: comparative biochemistry and genetics of the major plasma serpin. Comp. Biochem. Physiol. B Comp. Biochem. 100, 439–454. (doi:10.1016/0305-0491(91)90202-O) 10.1016/0305-0491(91)90202-o 1814672

[5] Silverman GA et al. 2001 The serpins are an expanding superfamily of structurally similar but functionally diverse proteins. Evolution, novel functions, mechanism of inhibition and a revised nomenclature. J. Biol. Chem. 276, 33 293–33 296. (doi:10.1074/jbc.R100016200) 10.1074/jbc.R100016200 11435447

[6] Davies GP, Lomas DA 2008 The molecular aetiology of the serpinopathies. Int. J. Biochem. Cell Biol. 40, 1273–1286. (doi:10.1016/j.biocel.2007.12.017) 18289918 10.1016/j.biocel.2007.12.017

[7] Irving JA et al. 2011 The serpinopathies: studying serpin polymerization *in vivo*. Meth. Enzymol. 501, 421–466. (doi:10.1016/B978-0-12-385950-1.00018-3) 22078544 10.1016/B978-0-12-385950-1.00018-3

[8] Lomas DA et al. 2005 Molecular mousetraps and the serpinopathies. Biochem. Soc. Trans. 33, 321–330. (doi:10.1042/BST0330321) 15787598 10.1042/BST0330321

[9] Lomas DA, Evans DL, Finch JT, Carrell RW 1992 The mechanism of Z alpha 1-antitrypsin accumulation in the liver. Nature 357, 605–607. (doi:10.1038/357605a0) 1608473 10.1038/357605a0

[10] Yamasaki M, Sendall TJ, Pearce MC, Whisstock JC, Huntington JA 2011 Molecular basis of *α*1-antitrypsin deficiency revealed by the structure of a domain-swapped trimer. EMBO Rep. 12, 1011–1017. (doi:10.1038/embor.2011.171) 21909074 10.1038/embor.2011.171 PMC3185345

[11] Davies RL et al. 2002 Association between conformational mutations in neuroserpin and onset and severity of dementia. Lancet 359, 2242–2247. (doi:10.1016/S0140-6736(02)09293-0) 12103288 10.1016/S0140-6736(02)09293-0

[12] Gettins PG 2002 Serpin structure, mechanism and function. Chem. Rev. 102, 4751–4804. (doi:10.1021/cr010170+) 12475206 10.1021/cr010170+

[13] Kaiserman D, Whisstock JC, Bird PI 2006 Mechanisms of serpin dysfunction in disease. Expert Rev. Mol. Med. 8, 1–19. (doi:10.1017/S1462399406000184) 17156576 10.1017/S1462399406000184

[14] Ligoxygakis P, Roth S, Reichhart JM 2004 A serpin regulates dorsal-ventral axis formation in the *Drosophila* embryo. Curr. Biol. 13, 2097–2102. (doi:10.1016/j.cub.2003.10.062) 14654000 10.1016/j.cub.2003.10.062

[15] Whisstock J, Skinner R, Lesk AM 1998 An atlas of serpin conformations. Trends Biochem. Sci. 23, 63–67. (doi:10.1016/S0968-0004(97)01172-9) 9538691 10.1016/s0968-0004(97)01172-9

[16] Huntington JA, Read RJ, Carrell RW 2000 Structure of a serpin-protease complex shows inhibition by deformation. Nature 407, 923–926. (doi:10.1038/35038119) 11057674 10.1038/35038119

[17] Ragg H, Lokot T, Kamp PB, Atchley WR, Dress A 2001 Vertebrate serpins: construction of a conflict-free phylogeny by combining exon-intron and diagnostic site analyses. Mol. Biol. Evol. 18, 577–584. (doi:10.1093/oxfordjournals.molbev.a003838) 11264410 10.1093/oxfordjournals.molbev.a003838

[18] Travis J, Salvesen G 1983 Control of coagulation and fibrinolysis by plasma proteinase inhibitors. Behring. Inst. Mitt. 73, 56–65. 6206838

[19] Patston PA 1995 Studies on inhibition of neutrophil cathepsin G by alpha 1-antichymotrypsin. Inflammation 19, 75–81. (doi:10.1007/BF01534382) 7705888 10.1007/BF01534382

[20] Matsumoto M, Tsuda M, Kusumi T, Takada S, Shimamura T, Katsunuma T 1981 Enhancement by *α*_1_-antichymotrypsin of antibody response *in vivo*. Biochem. Biophys. Res. Commun. 100, 478–482. (doi:10.1016/S0006-291X(81)80121-0) 7020696 10.1016/s0006-291x(81)80121-0

[21] Gravagna P, Gianazza E, Arnaud P, Neels M, Ades EW 1982 Modulation of the immune response by plasma protease inhibitors. III. α_1_-Antichymotrypsin inhibits human natural killing and antibody-dependent cell-mediated cytotoxicity. J. Reticuloendothelial Soc. 32, 125–130. 6897659

[22] Belorgey D, Hägglöf P, Karisson-Li S, Lomas DA 2007 Protein misfolding and the serpinopathies. Prion 1, 15–20. (doi:10.4161/pri.1.1.3974) 19164889 10.4161/pri.1.1.3974 PMC2633702

[23] Dou C, Zhang J, Sun Y, Zhao X, Wu Q, Ji C, Gu S, Xie Y, Mao Y 2013 The association of ACT-17 A/T polymorphism with Alzheimer's disease: a meta-analysis. Curr. Alzheimer Res. 10, 63–71. (doi:10.2174/1567205011310010009) 22272609

[24] Billingsley GD, Walter MA, Hammond GL, Cox DW 1993 Physical mapping of four serpin genes: α1-antitrypsin, α1-antichymotrypsin, corticosteroid-binding globulin, and protein C inhibitor within a 280-kb region on chromosome 14q32.1. Am. J. Hum. Genet. 52, 343–353. 8381582 PMC1682208

[25] Forsyth S, Horvath A, Coughlin P 2003 A review and comparison of the murine *α*_1_-antitrypsin and *α*_1_-antichymotrypsin multi-gene clusters with the human clade A serpins. Genomics 81, 336–345. (doi:10.1016/S0888-7543(02)00041-1) 12659817 10.1016/s0888-7543(02)00041-1

[26] Stratil A, Cizova-Schröffelova D, Gabrisova E, Pavlik M, Coppieters W, Peelman L, Van de Weghe A, Bouquet Y 1995 Pig plasma α-protease inhibitors PI2, PI3 and PI4 are members of the antichymotrypsin family. Comp. Biochem. Physiol. B. 111, 53–60. (doi:10.1016/0305-0491(94)00232-J) 7749636 10.1016/0305-0491(94)00232-j

[27] Stratil A, Peelman LJ, Mattheeuws M, Van Poucke M, Reiner G, Geldermann H 2002 A novel porcine gene, α-1-antichymotrypsin 2 (*SERPINA3-2*): sequence, genomic organization, polymorphism and mapping. Gene 292, 113–119. (doi:10.1016/S0378-1119(02)00665-0) 12119105 10.1016/s0378-1119(02)00665-0

[28] Pélissier P, Delourme D, Germot A, Blanchet X, Becila S, Maftah A, Leveziel H, Ouali A, Brémaud L 2008 An original *SERPINA3* gene cluster: elucidation of genomic organization and gene expression in the *Bos taurus* 21q24 region. BMC Genomics 9, 151 (doi:10.1186/1471-2164-9-151) 18384666 10.1186/1471-2164-9-151 PMC2373789

[29] Hwang SR, Steineckert B, Yasothornsrikul S, Sei CA, Toneff T, Rattan J, Hook VY 1999 Molecular cloning of endopin 1, a novel serpin localized to neurosecretory vesicles of chromaffin cells. J. Biol. Chem. 274, 34 164–34 173. (doi:10.1074/jbc.274.48.34164) 10.1074/jbc.274.48.34164 10567388

[30] Hwang SR, Steineckert B, Toneff T, Bundey R, Logvinova AV, Goldsmith P, Hook VY 2002 The novel serpin endopin 2 demonstrates cross-class inhibition of papain and elastase: localization of endopin 2 to regulated secretory vesicles of neuroendocrine chromaffin cells. Biochemistry 41, 10 397–10 405. (doi:10.1021/bi020088o) 10.1021/bi020088o 12173926

[31] Hwang S-R, Bundey R, Toneff T, Hook V 2009 Endopin serpin protease inhibitors localize with neuropeptides in secretory vesicles and neuroendocrine tissues. Neuroendocrinology 89, 210–216. (doi:10.1159/000162916) 18840998 10.1159/000162916 PMC2731708

[32] Tassy C et al. 2005 Muscle endopin 1, a muscle intracellular serpin which strongly inhibits elastase: purification, characterization, cellular localization and tissue distribution. Biochem. J. 388, 273–280. (doi:10.1042/BJ20041921) 15647007 10.1042/BJ20041921 PMC1186716

[33] Brémaud L et al. 2006 Purification of the skeletal muscle protein Endopin 1B and characterization of the genes encoding Endopin 1A and 1B isoforms. FEBS Lett. 580, 3477–3484. (doi:10.1016/j.febslet.2006.04.099) 16716310 10.1016/j.febslet.2006.04.099

[34] Herrera-Mendez CH et al. 2009 Inhibition of human initiator caspase 8 and effector caspase 3 by cross-class inhibitory bovSERPINA3-1 and A3-3. FEBS Lett. 583, 2743–2748. (doi:10.1016/j.febslet.2009.07.055) 19665028 10.1016/j.febslet.2009.07.055

[35] Boudida Y, Gagaoua M, Becila S, Picard B, Boudjellal A, Herrera-Mendez CH, Sentandreu M, Ouali A In press. Serine protease inhibitors as good predictors of meat tenderness: which are they and what are their functions? Crit. Rev. Food. Sci. Nutr. (doi:10.1080/10408398.2012.741630) 10.1080/10408398.2012.741630 25085261

[36] Blanchet X et al. 2012 Mutagenesis of the bovSERPINA3-3 demonstrates the requirement of aspartate-371 for inter-molecular interaction and formation of dimers. Protein Sci. 21, 977–986. (doi:10.1002/pro.2078) 22505318 10.1002/pro.2078 PMC3403435

[37] Yamasaki M, Li W, Johnson DJ, Huntington JA 2008 Crystal structure of a stable dimer reveals the molecular basis of serpin polymerization*.* Nature 455, 1255–1258. (doi:10.1038/nature07394) 18923394 10.1038/nature07394

[38] Tarentino AL, Gomez CM, Plummer TH 1985 Deglycosylation of asparagine-linked glycans by peptide: *N*-glycosydase F. Biochemistry 24, 4665–4671. (doi:10.1021/bi00338a028) 4063349 10.1021/bi00338a028

[39] Jensen PH, Kolarich D, Packer NH 2010 Mucin-type *O*-glycosylation--putting the pieces together. FEBS J. 277, 81–94. (doi:10.1111/j.1742-4658.2009.07429.x) 19919547 10.1111/j.1742-4658.2009.07429.x

[40] Kalsheker N, Morley S, Morgan K 2002 Gene regulation of the serine proteinase inhibitors α1-antitrypsin and α1-antichymotrypsin. Biochem. Soc. Trans. 30, 93–98. (doi:10.1042/bst0300093) 12023832 10.1042/

[41] Hoffmann DC et al. 2011 Pivotal role of α1-antichymotrypsin in skin repair. J. Biol. Chem. 219, 28 889–28 901. (doi:10.1074/jbc.M111.249979) 10.1074/jbc.M111.249979 PMC3190696 21693707

[42] Sun W et al. 2011 *N*-Glycans of human protein C inhibitor: tissue-specific expression and function. PLoS ONE 6, e29011 (doi:10.1371/journal.pone.0029011) 22205989 10.1371/journal.pone.0029011 PMC3242763

[43] Avvakumov GV, Strel'chyonok OA 1988 Evidence for the involvement of the transcortin carbohydrate moiety in the glycoprotein interaction with the plasma membrane of human placental syncytiotrophoblast. Biochim. Biophys. Acta 938, 1–6. (doi:10.1016/0005-2736(88)90115-0) 3337811 10.1016/0005-2736(88)90115-0

[44] Strel'chyonok OA, Avvakumov GV 1990 Specific steroid-binding glycoproteins of human blood plasma: novel data on their structure and function. J. Steroid Biochem. 35, 519–534. (doi:10.1016/0022-4731(90)90195-X) 2192196 10.1016/0022-4731(90)90195-x

[45] Sumer-Bayraktar Z, Kolarich D, Campbell MP, Ali S, Packer NH, Thaysen-Andersen M 2011 *N*-Glycans modulate the function of human corticosteroid-binding globulin. Mol. Cell Proteomics 10, M111009100. (doi:10.1074/mcp.M111.009100) 21558494 10.1074/mcp.M111.009100 PMC3149095

[46] Eppenberger HM, Dawson DM, Kaplan NO 1967 The comparative enzymology of creatine kinases. I. Isolation and characterization from chicken and rabbit tissues. J. Biol. Chem. 242, 204–209. 6016604

[47] Winnard P, Cashon RE, Sidell BD, Vayda ME 2003 Isolation, characterization and nucleotide sequence of the muscle isoforms of creatine kinase from the Antarctic teleost *Chaenocephalus aceratus*. Comp. Biochem. Physiol. B Biochem. Mol. Biol. 134, 651–667. (doi:10.1016/S1096-4959(03)00025-3) 12670791 10.1016/s1096-4959(03)00025-3

[48] Wallimann T, Schlösser T, Eppenberger HM 1984 Function of M-line-bound creatine kinase as intramyofibrillar ATP regenerator at the receiving end of the phosphorylcreatine shuttle in muscle. J. Biol. Chem. 259, 5238–5246. 6143755

[49] Hornemann T, Kempa S, Himmel M, Hayess K, Fürst DO, Wallimann T 2003 Muscle-type creatine kinase interacts with central domains of the M-band proteins myomesin and M-protein. J. Mol. Biol. 332, 877–887. (doi:10.1016/S0022-2836(03)00921-5) 12972258 10.1016/s0022-2836(03)00921-5

[50] Okumura N, Hashida-Okumura A, Kita K, Matsubae M, Matsubara T, Takao T, Nagai K 2005 Proteomic analysis of slow- and fast-twitch skeletal muscles. Proteomics 5, 2896–2906. (doi:10.1002/pmic.200401181) 15981298 10.1002/pmic.200401181

[51] Hu WJ, Zhou SM, Yang JS, Meng FG 2011 Computational simulations to predict creatine kinase-associated factors: protein–protein interaction studies of brain and muscle types of creatine kinases. Enzyme Res. 2011, 328249 (doi:10.4061/2011/328249) 21826261 10.4061/2011/328249 PMC3150154

[52] Simonovic M, Gettins PG, Volz K 2001 Crystal structure of human PEDF, a potent anti-angiogenic and neurite growth-promoting factor. Proc Natl Acad Sci USA 98, 11 131–11 135. (doi:10.1073/pnas.211268598) 11562499 10.1073/pnas.211268598 PMC58695

[53] Bradford MM 1976 A rapid and sensitive method for the quantitation of microgram quantities of protein utilizing the principle of protein–dye binding. Anal. Biochem. 72, 248–254. (doi:10.1016/0003-2697(76)90527-3) 942051 10.1016/0003-2697(76)90527-3

[54] Laemmli UK 1970 Cleavage of structural proteins during the assembly of the head of bacteriophage T4. Nature 277, 680–685. (doi:10.1038/227680a0) 5432063 10.1038/227680a0

[55] Dutaud D, Aubry L, Henry L, Levieux D, Hendil KB, Kueh LN, Bureau JP, Ouali A 2002 Development and evaluation of a sandwich ELISA for quantification of the 20S proteasome in human plasma. J. Immunol. Met. 260, 183–193. (doi:10.1016/S0022-1759(01)00555-5) 10.1016/s0022-1759(01)00555-5 11792388

[56] Beatty K, Bieth J, Travis J 1980 Kinetics of association of serine proteinases with native and oxidized *α*-proteinase inhibitor and *α*-1-antichymotrypsin. J. Biol. Chem. 255, 3931–3934. 6989830

[57] Shevchenko A, Tomas H, Havlis J, Olsen JV, Mann M 2006 In-gel digestion for mass spectrometric characterization of proteins and proteomes. Nat. Protoc. 1, 2856–2860. (doi:10.1038/nprot.2006.468) 17406544 10.1038/nprot.2006.468

