# Supplementary material for: Expression of SERPINA3s in cattle: focus on bovSERPINA3-7 reveals specific involvement in skeletal muscle
Source: Open Biol. 2015 Sep 9;5(9):150071. doi: 10.1098/rsob.150071 (PMC4593666; doi:10.1098/rsob.150071)
Supplement: Table S1. Table S2. Table S3. Table S4. Figure S1 [file rsob150071supp1.docx]

**Supplementary material**

Table S1: Ct values of qPCR from tissues analysis performed on 3 pooled samples.

Each value is the mean of three measurements. N.D. (not detected).

____________________________________________________________________________________________________________

Ct values ± S.D.

____________________________________________________________________________________________________________

*SA3-1 SA3-3/4 SA3-5 SA3-6 SA3-7 SA3-8 TFIID*

liver 19.64 ± 0.01 18.47 ± 0.03 17.96 ± 0.03 20.74 ± 0.05 19.05 ± 0.02 23.29 ± 0.04 27.99±0.04

lung 33.67 ± 0.07 31.97 ± 0.05 32.77 ± 0.09 33.33 ± 0.11 N.D. N.D. 27.40±0.03

testis 27.04 ± 0.03 33.05 ± 0.17 36.08 ± 0.10 35.25 ± 0.03 35.31 ± 0.06 35.98 ± 0.29 23.22±0.07

kidney 29.16 ± 0.04 27.55 ± 0.03 28.24 ± 0.03 28.96 ± 0.03 22.01 ± 0.15 38.78 ± 0.10 31.71±0.07

spleen 29.44 ± 0.08 27.76 ± 0.05 28.60 ± 0.03 28.99 ± 0.06 37.45 ± 0.18 34.84 ± 0.08 27.48±0.10

thymus 34.29 ± 0.05 32.32 ± 0.06 33.75 ± 0.04 34.99 ± 0.04 36.04 ± 0.08 35.20 ± 0.00 24.92±0.06

cerebellum 31.72 ± 0.01 29.39 ± 0.03 31.06 ± 0.06 31.41 ± 0.03 34.97 ± 0.01 35.61 ± 0.37 28.16±0.09

skeletal muscle 32.69 ± 0.11 31.10 ± 0.16 31.86 ± 0.51 32.80 ± 0.01 38.92 ± 0.03 36.54 ± 0.07 28.28±0.05

Table S2: RQ values calculated from Ct, normalized by TFIID transcript and calibrated by *bovSERPINA3* transcripts in testis. N.D. (Not Detected).

_________________________________________________________________________________________________________________

RQ values ± S.D.

_________________________________________________________________________________________________________________

*SA3-1 SA3-3/4 SA3-5 SA3-6 SA3-7 SA3-8*

liver 3686.19 ±95.39 502843.41±71998.57 3996289.45±609770.32 519477.04±37252.01 2526762.51±715992.31 154192.02±19133.55

lung 0.11±0.00 32.42±5.00 117.91±14.67 66.71±4.15 N.D. N.D.

testis 1.00±0.00 1.00±0.00 1.00±0.00 1.00±0.00 1.00±0.00 1.00±0.00

kidney 59.14±3.97 13373.78±1368.42 50536.38±723.76 18834.66±723.76 52.31±12.38 534.47±62.93

spleen 1.47±0.02 315.83±21.68 1107.19±203.67 469.94±18.00 3.89±0.44 29.58±1.12

thymus 0.02±0.00 5.07±1.08 16.37±1.41 4.93±0.78 2.17±0.63 4.93±1.27

cerebellum 0.44±0.05 139.31±13.62 389.89±44.92 207.45±15.31 52.51±13.1 43.61±15.79

skeletal muscle 0.79±0.08 155.37±22.88 662.53±65.27 217.96±7.67 4.35±1.19 26.78±9.52

Table S3: Proportions of transcripts of *bovSERPINA3* genes in each tissue. N.D. (Not Detected).

____________________________________________________________________________________________________________

Percentages of transcript values ± S.D.

____________________________________________________________________________________________________________

*SA3-1 SA3-3/4 SA3-5 SA3-6 SA3-7 SA3-8*

liver 13.90%±0.23% 27.68%±1.18% 29.00%±1.24% 6.03%±0.38% 22.22%±0.62% 1.18%±0.05%

lung 10.90%±0.66% 46.51%±1.31% 22.40%±1.19% 20.20%±1.09% N.D. N.D.

testis 97.63%±0.08% 1.44%±0.14% 0.19%±0.03% 0.30%±0.00% 0.24%±0.06% 0.20%±0.01%

kidney 14.36%±0.27% 47.61%±1.14% 23.65%±0.88% 14.08%±0.13% 0.03%±0.00% 0.26%±0.01%

spleen 15.12%±0.66% 47.52%±1.29% 21.81%±1.64% 14.83%±0.57% 0.10%±0.04% 0.62%±0.02%

thymus 12.75%±0.67% 47.37%±1.63% 20.46%±1.11% 9.72%±0.52% 3.34%±0.90% 6.35%±0.70%

cerebellum 10.66%±1.10% 50.02%±0.70% 18.46%±0.82% 15.64%±0.38% 3.02%±0.19% 2.20%±0.91%

skeletal muscle 15.59%±0.84% 44.67%±0.57% 25.24%±0.82% 13.22%±0.35% 0.20%±0.02% 1.08%±0.41%

Table S4: Mass spectrometry analysis by LTQ-Orbitrap.

| **Compar_Spectra** |  |  |  |  |  |  |  |
| --- | --- | --- | --- | --- | --- | --- | --- |
| Description | Redundancy | Coverage | MW | Evalue | Sample_E01 | Sample_E02 |  |
| sp\|Q9XSC6\|KCRM_BOVIN Creatine kinase M-type OS=Bos taurus | - | 61 | 42.9 | -68.8 | 34 | 15 |  |
|  |  |  |  |  |  |  |  |
| **Compar_Specific** |  |  |  |  |  |  |  |
| Description | Redundancy | Coverage | MW | Evalue | Sample_E01 | Sample_E02 |  |
| sp\|Q9XSC6\|KCRM_BOVIN Creatine kinase M-type OS=Bos taurus | - | 61 | 42.9 | -68.8 | 34 | 15 |  |
|  |  |  |  |  |  |  |  |
| **Compar_Unique** |  |  |  |  |  |  |  |
| Description | Redundancy | Coverage | MW | Evalue | Sample_E01 | Sample_E02 |  |
| sp\|Q9XSC6\|KCRM_BOVIN Creatine kinase M-type OS=Bos taurus | - | 61 | 42.9 | -68.8 | 20 | 11 |  |
|  |  |  |  |  |  |  |  |
| **Compar_PAI** |  |  |  |  |  |  |  |
| Description | Redundancy | Coverage | MW | Evalue | Sample_E01 | Sample_E02 |  |
| sp\|Q9XSC6\|KCRM_BOVIN Creatine kinase M-type OS=Bos taurus | - | 61 | 42.9 | -68.8 | 2.333 | 1.000 |  |
|  |  |  |  |  |  |  |  |
| **Proteins** |  |  |  |  |  |  |  |
| Description | log(E value) | Coverage | MW | Spectra | Specific uniques | Uniques | PAI |
| sp\|Q9XSC6\|KCRM_BOVIN Creatine kinase M-type OS=Bos taurus | -68.76988983 | 61 | 42.9 | 56 | - | 23 | 3.111111164 |

| **Peptides** |  |  |  |  |  |  |  |  |  |  |  |  |  |
| --- | --- | --- | --- | --- | --- | --- | --- | --- | --- | --- | --- | --- | --- |
| Description | Sample | Scan | Rt | Sequence | Modifs | Used | on a total of | E-value | Charge | MH+ Obs | MH+ theo | DeltaMH+ | Delta-ppm |
| sp\|Q9XSC6\|KCRM_BOVIN crea | Sample_E01 | 490 | 8.170000076 | HGGFKPTDK |  | - | - | 2.5E-05 | 2 | 986.504517 | 986.505798 | -0.0013 | -1.3177824 |
| sp\|Q9XSC6\|KCRM_BOVIN crea | Sample_E01 | 491 | 8.170000076 | PFGNTHNK |  | - | - | 0.0026 | 2 | 914.447083 | 914.448303 | -0.0012 | -1.3122667 |
| sp\|Q9XSC6\|KCRM_BOVIN crea | Sample_E01 | 511 | 8.279999733 | HKTDLNHENLK |  | - | - | 0.0026 | 2 | 1348.69897 | 1348.69714 | 0.0018 | 1.33462131 |
| sp\|Q9XSC6\|KCRM_BOVIN crea | Sample_E01 | 609 | 8.840000153 | TDLNHENLK |  | - | - | 0.00038 | 2 | 1083.54272 | 1083.54333 | -0.0006 | -0.5537388 |
| sp\|Q9XSC6\|KCRM_BOVIN crea | Sample_E01 | 624 | 8.930000305 | GGNMKEVFR | M4:+15.99491 | - | - | 0.049 | 3 | 1053.5127 | 1053.51501 | -0.0023 | -2.1831679 |
| sp\|Q9XSC6\|KCRM_BOVIN crea | Sample_E01 | 694 | 9.340000153 | LLASGMAR | M6:+15.99491 | - | - | 0.0047 | 2 | 834.4505 | 834.450623 | -1E-04 | -0.1198393 |
| sp\|Q9XSC6\|KCRM_BOVIN crea | Sample_E01 | 799 | 9.960000038 | GYALPPHCSR | C8:+57.02146 | - | - | 0.037 | 3 | 1157.55078 | 1157.55249 | -0.0017 | -1.4686159 |
| sp\|Q9XSC6\|KCRM_BOVIN crea | Sample_E01 | 801 | 9.970000267 | GYALPPHCSR | C8:+57.02146 | - | - | 0.0033 | 2 | 1157.5509 | 1157.55249 | -0.0015 | -1.2958376 |
| sp\|Q9XSC6\|KCRM_BOVIN crea | Sample_E01 | 821 | 10.09000015 | PPHCSR | C4:+57.02146 | - | - | 0.0022 | 2 | 753.342834 | 753.346497 | -0.0037 | -4.9114189 |
| sp\|Q9XSC6\|KCRM_BOVIN crea | Sample_E01 | 847 | 10.23999977 | TDLNHENLK |  | - | - | 0.00072 | 2 | 1083.54333 | 1083.54333 | 0 | 0 |

| sp\|Q9XSC6\|KCRM_BOVIN crea | Sample_E01 | 913 | 10.64000034 | GYALPPHCSR | C8:+57.02146 | - | - | 0.005 | 2 | 1157.55042 | 1157.55249 | -0.002 | -1.7277834 |
| --- | --- | --- | --- | --- | --- | --- | --- | --- | --- | --- | --- | --- | --- |
| sp\|Q9XSC6\|KCRM_BOVIN crea | Sample_E01 | 1025 | 11.32999992 | GYALPPHCSR | C8:+57.02146 | - | - | 0.00017 | 2 | 1157.5542 | 1157.55249 | 0.0018 | 1.55500507 |
| sp\|Q9XSC6\|KCRM_BOVIN crea | Sample_E01 | 1143 | 12.02999973 | GYALPPHCSR | C8:+57.02146 | - | - | 0.0027 | 3 | 1157.55176 | 1157.55249 | -0.0007 | -0.6047242 |
| sp\|Q9XSC6\|KCRM_BOVIN crea | Sample_E01 | 1290 | 12.89999962 | GQSIDDMIPAQK | M7:+15.99491 | - | - | 0.0051 | 2 | 1318.63147 | 1318.63123 | 0.0004 | 0.30334485 |
| sp\|Q9XSC6\|KCRM_BOVIN crea | Sample_E01 | 1374 | 13.40999985 | AEEEYPDLSK |  | - | - | 0.00016 | 2 | 1180.53699 | 1180.53735 | -0.0002 | -0.1694144 |
| sp\|Q9XSC6\|KCRM_BOVIN crea | Sample_E01 | 1444 | 13.82999992 | TDLNHENLK |  | - | - | 0.0095 | 2 | 1083.5415 | 1083.54333 | -0.0018 | -1.6612164 |
| sp\|Q9XSC6\|KCRM_BOVIN crea | Sample_E01 | 1684 | 15.27000046 | FCVGLQK | C2:+57.02146 | - | - | 0.0032 | 2 | 851.444641 | 851.444824 | -0.0002 | -0.2348948 |
| sp\|Q9XSC6\|KCRM_BOVIN crea | Sample_E01 | 1719 | 15.47999954 | AEEEYPDLSK |  | - | - | 0.0037 | 2 | 1180.53784 | 1180.53735 | 0.0006 | 0.50824314 |
| sp\|Q9XSC6\|KCRM_BOVIN crea | Sample_E01 | 1720 | 15.47999954 | GYALPPHCSR | C8:+57.02146 | - | - | 0.001 | 3 | 1157.55261 | 1157.55249 | 1E-04 | 0.08638917 |
| sp\|Q9XSC6\|KCRM_BOVIN crea | Sample_E01 | 1913 | 16.64999962 | ALTLEIYKK |  | - | - | 0.023 | 2 | 1078.65125 | 1078.65112 | 0.0002 | 0.18541676 |
| sp\|Q9XSC6\|KCRM_BOVIN crea | Sample_E01 | 2159 | 18.14999962 | FCVGLQK | C2:+57.02146 | - | - | 0.04 | 2 | 851.44397 | 851.444824 | -0.0008 | -0.9395794 |
| sp\|Q9XSC6\|KCRM_BOVIN crea | Sample_E01 | 2292 | 18.96999931 | LNFKAEEEYPDLSK |  | - | - | 0.002 | 3 | 1682.828 | 1682.82764 | 0.0004 | 0.23769516 |
| sp\|Q9XSC6\|KCRM_BOVIN crea | Sample_E01 | 3666 | 27.62000084 | FEEILTR |  | - | - | 0.019 | 2 | 907.489868 | 907.48877 | 0.0011 | 1.21213627 |
| sp\|Q9XSC6\|KCRM_BOVIN crea | Sample_E01 | 3812 | 28.54999924 | GTGGVDTAAVGSVFD |  | - | - | 0.028 | 2 | 1352.63403 | 1352.6333 | 0.0008 | 0.59143895 |
| sp\|Q9XSC6\|KCRM_BOVIN crea | Sample_E01 | 3944 | 29.38999939 | SFLVWVNEEDHLR |  | - | - | 2.6E-05 | 2 | 1643.81726 | 1643.81812 | -0.0007 | -0.4258378 |
| sp\|Q9XSC6\|KCRM_BOVIN crea | Sample_E01 | 4000 | 29.75 | LGSSEVEQVQLVVDGVK |  | - | - | 0.014 | 2 | 1785.95813 | 1785.95972 | -0.0015 | -0.8398846 |
| sp\|Q9XSC6\|KCRM_BOVIN crea | Sample_E01 | 4462 | 32.72999954 | DLFDPIIQDR |  | - | - | 0.0037 | 2 | 1231.63232 | 1231.6322 | 0.0002 | 0.16238615 |
| sp\|Q9XSC6\|KCRM_BOVIN crea | Sample_E01 | 4539 | 33.22000122 | LSVEALNSLTGEFK |  | - | - | 0.00073 | 2 | 1507.79858 | 1507.80066 | -0.002 | -1.3264353 |
| sp\|Q9XSC6\|KCRM_BOVIN crea | Sample_E01 | 4574 | 33.43999863 | DLFDPIIQDR |  | - | - | 9.4E-05 | 2 | 1231.63269 | 1231.6322 | 0.0006 | 0.48715845 |
| sp\|Q9XSC6\|KCRM_BOVIN crea | Sample_E01 | 4785 | 34.83000183 | DLFDPIIQDR |  | - | - | 0.0057 | 2 | 1231.63306 | 1231.6322 | 0.001 | 0.81193078 |
| sp\|Q9XSC6\|KCRM_BOVIN crea | Sample_E01 | 4798 | 34.90999985 | ETPSGFTLDDVIQTGVDN  PGHPFIMTVGCVAGDEES  YTVFK | C29:+57.02146 | - | - | 0.0034 | 3 | 4430.05322 | 4430.04834 | 0.005 | 1.12865591 |
| sp\|Q9XSC6\|KCRM_BOVIN crea | Sample_E01 | 4854 | 35.27000046 | LSVEALNSLTGEFK |  | - | - | 0.036 | 2 | 1507.80017 | 1507.80066 | -0.0005 | -0.3316088 |
| sp\|Q9XSC6\|KCRM_BOVIN crea | Sample_E01 | 4896 | 35.52999878 | DLFDPIIQDR |  | - | - | 0.022 | 2 | 1231.63208 | 1231.6322 | 0 | 0 |
| sp\|Q9XSC6\|KCRM_BOVIN crea | Sample_E01 | 5120 | 36.95000076 | DLFDPIIQDR |  | - | - | 0.0035 | 2 | 1231.63281 | 1231.6322 | 0.0007 | 0.56835151 |
| sp\|Q9XSC6\|KCRM_BOVIN crea | Sample_E02 | 595 | 8.140000343 | HGGFKPTDK |  | - | - | 0.00018 | 2 | 986.504395 | 986.505798 | -0.0014 | -1.4191504 |
| sp\|Q9XSC6\|KCRM_BOVIN crea | Sample_E02 | 596 | 8.149999619 | PFGNTHNK |  | - | - | 0.032 | 2 | 914.446777 | 914.448303 | -0.0016 | -1.7496887 |
| sp\|Q9XSC6\|KCRM_BOVIN crea | Sample_E02 | 699 | 8.75 | TDLNHENLK |  | - | - | 0.0038 | 2 | 1083.54272 | 1083.54333 | -0.0006 | -0.5537388 |
| sp\|Q9XSC6\|KCRM_BOVIN crea | Sample_E02 | 926 | 10.11999989 | PPHCSR | C4:+57.02146 | - | - | 0.036 | 2 | 753.346985 | 753.346497 | 0.0005 | 0.66370523 |
| sp\|Q9XSC6\|KCRM_BOVIN crea | Sample_E02 | 1006 | 10.60999966 | GYALPPHCSR | C8:+57.02146 | - | - | 0.0041 | 2 | 1157.552 | 1157.55249 | -0.0004 | -0.3455567 |
| sp\|Q9XSC6\|KCRM_BOVIN crea | Sample_E02 | 1013 | 10.64999962 | GYALPPHCSR | C8:+57.02146 | - | - | 0.0023 | 3 | 1157.55249 | 1157.55249 | 0 | 0 |
| sp\|Q9XSC6\|KCRM_BOVIN crea | Sample_E02 | 1120 | 11.28999996 | GYALPPHCSR | C8:+57.02146 | - | - | 0.041 | 2 | 1157.55286 | 1157.55249 | 0.0004 | 0.34555668 |
| sp\|Q9XSC6\|KCRM_BOVIN crea | Sample_E02 | 1127 | 11.34000015 | GYALPPHCSR | C8:+57.02146 | - | - | 0.028 | 3 | 1157.55151 | 1157.55249 | -0.001 | -0.8638917 |
| sp\|Q9XSC6\|KCRM_BOVIN crea | Sample_E02 | 1341 | 12.64000034 | GQSIDDMIPAQK | M7:+15.99491 | - | - | 0.005 | 2 | 1318.63123 | 1318.63123 | 1E-04 | 0.07583621 |

| sp\|Q9XSC6\|KCRM_BOVIN crea | Sample_E02 | 1466 | 13.39999962 | AEEEYPDLSK |  | - | - | 0.00023 | 2 | 1180.5376 | 1180.53735 | 0.0004 | 0.33882874 |
| --- | --- | --- | --- | --- | --- | --- | --- | --- | --- | --- | --- | --- | --- |
| sp\|Q9XSC6\|KCRM_BOVIN crea | Sample_E02 | 3185 | 24.42000008 | GGDDLDPNYVLSSR |  | - | - | 0.031 | 2 | 1507.70386 | 1507.70276 | 0.0012 | 0.79591292 |
| sp\|Q9XSC6\|KCRM_BOVIN crea | Sample_E02 | 3688 | 27.75 | GTGGVDTAAVGSVFDVS  NADR |  | - | - | 0.01 | 2 | 1994.9436 | 1994.94177 | 0.0018 | 0.90228194 |
| sp\|Q9XSC6\|KCRM_BOVIN crea | Sample_E02 | 4070 | 30.28000069 | LGSSEVEQVQLVVDGVK |  | - | - | 0.019 | 3 | 1785.96033 | 1785.95972 | 0.0007 | 0.39194611 |
| sp\|Q9XSC6\|KCRM_BOVIN crea | Sample_E02 | 4491 | 33.08000183 | DLFDPIIQDR |  | - | - | 0.0068 | 2 | 1231.63245 | 1231.6322 | 0.0003 | 0.24357922 |
| sp\|Q9XSC6\|KCRM_BOVIN crea | Sample_E02 | 4825 | 35.24000168 | DLFDPIIQDR |  | - | - | 0.038 | 2 | 1231.63196 | 1231.6322 | -1E-04 | -0.0811931 |

Figure S1: Workflow of experiments.
